# Synthesis and Degradation of Vinyl Polymers with Evenly Distributed Thioacetal Bonds in Main Chains: Cationic DT Copolymerization of Vinyl Ethers and Cyclic Thioacetals

**DOI:** 10.1002/anie.202215021

**Published:** 2022-12-07

**Authors:** Mineto Uchiyama, Yukihiro Murakami, Kotaro Satoh, Masami Kamigaito

**Affiliations:** ^1^ Department of Molecular and Macromolecular Chemistry Graduate School of Engineering Nagoya University Furo-cho, Chikusa-ku, Nagoya 464-8603 Japan; ^2^ Department of Chemical Science and Engineering School of Materials and Chemical Technology Tokyo Institute of Technology 2-12-1-H120 Ookayama Meguro-ku, Tokyo 152-8550 Japan

**Keywords:** Cationic Polymerization, Degradable Polymer, Living Polymerization, Thioacetal, Vinyl Ether

## Abstract

We report a novel method to synthesize degradable poly(vinyl ether)s with cleavable thioacetal bonds periodically arranged in the main chains using controlled cationic copolymerization of vinyl ethers with a 7‐membered cyclic thioacetal (**7‐CTA**) via degenerative chain transfer (DT) to the internal thioacetal bonds. The thioacetal bonds, which are introduced into the main chain by cationic ring‐opening copolymerization of **7‐CTA** with vinyl ethers, serve as in‐chain dormant species to allow homogeneous propagation of vinyl ethers for all internal segments to afford copolymers with controlled overall and segmental molecular weights. The obtained polymers can be degraded into low‐ and controlled‐molecular‐weight polymers with narrow molecular weight distributions via hydrolysis. Various vinyl ethers with hydrophobic, hydrophilic, and functional pendants are available. Finally, one‐pot synthesis of multiblock copolymers and their degradation into diblock copolymers are also achieved.

## Graphical abstract

Degradable poly(vinyl ether)s were synthesized with periodically arranged in‐chain thioacetal bonds via cationic degenerative chain‐transfer copolymerization of vinyl ethers with a 7‐membered cyclic thioacetal. The copolymers can be degraded into low‐ and controlled‐molecular‐weight polymers via hydrolysis. One‐pot synthesis of multiblock copolymers and their degradation into diblock copolymers is also achieved.

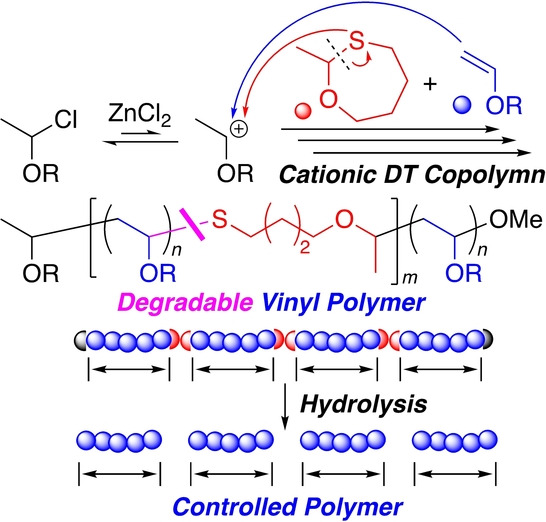

## Introduction

Furnishing synthetic polymers with appropriate degradability is one of the most important topics in chemistry as a solution to global environmental issues caused by plastic waste.[^1^, ^2^, ^3^, ^4^, ^5^] In particular, this is highly challenging for vinyl polymers, which are robust but difficult to degrade due to their stable carbon‐carbon backbones. To overcome this issue, various strategies for providing or triggering degradation to vinyl polymers have been proposed.[^6^, ^7^, ^8^, ^9^, ^10^, ^11^, ^12^, ^13^, ^14^, ^15^, ^16^, ^17^, ^18^, ^19^, ^20^, ^21^, ^22^] Among them, copolymerization of heteroatom‐containing cyclic monomers with common vinyl monomers, by which labile carbon‐heteroatom bonds can be introduced via ring opening of the cyclic structures, is one of the most efficient methods.[^23^, ^24^, ^25^, ^26^] Various cyclic compounds, which are copolymerizable via ring‐opening reactions, have been developed to date and include cyclic ketene acetals,[^27^, ^28^, ^29^, ^30^, ^31^, ^32^, ^33^, ^34^, ^35^, ^36^, ^37^, ^38^, ^39^, ^40^] thionolactones,[^41^, ^42^, ^43^, ^44^, ^45^, ^46^, ^47^] and cyclic allylic sulfur compounds[^48^, ^49^, ^50^, ^51^, ^52^] for radical polymerization as well as oxiranes,[^53^, ^54^] cyclic acetals,[^55^, ^56^] hemiacetal esters,^[57]^ and 1,3‐dioxa‐2‐silacycloalkanes^[58]^ for cationic polymerization.

However, incorporation of the cyclic monomers in the resulting copolymers depends on monomer reactivity ratios to the vinyl comonomers, and thus, the distributions of degradable carbon‐heteroatom bonds in the main chain are not homogeneous except for extreme cases such as alternating copolymerizations. Namely, the segmental lengths between the degradable units are not arbitrarily controlled in general. The degradation of such heterogeneous copolymers results in heterogeneity in the lengths or sizes of the degraded polymer or oligomer chains. In view of further potential biological degradation of the resulting oligomers through cell walls under natural environments, controlled degraded polymer chain lengths are more desirable. Therefore, homogeneous incorporation of heteroatoms into the main chain, which enables precise degradation, is the next challenging target for designing degradable vinyl polymers.

Recently, we developed novel precision cationic polymerizations that proceed via degenerative chain‐transfer (DT) mechanisms through reversible activation of carbon‐sulfur bonds and enable excellent control of polymer chain lengths over a wide range of molecular weights (*M*_n_=10^3^–10^5^) with narrow molecular weight distributions (*M*_w_/*M*_n_≈1.1).[^59^, ^60^, ^61^, ^62^, ^63^, ^64^, ^65^] In particular, thioacetals or thioethers serve as efficient and reversible chain‐transfer agents for the cationic polymerization of vinyl ethers and styrene derivatives and are relatively stable and easy to handle even in air.[^60^, ^64^, ^65^, ^66^, ^67^, ^68^, ^69^, ^70^, ^71^, ^72^] In this cationic polymerization, the vinyl monomers are formally inserted into the terminal C−S bonds derived from the sulfur compounds via reversible generation of the propagating cationic species based on the DT mechanism. The fast interconversion between the dormant C−S species and the propagating cationic species enables the precise control of polymer chain lengths.

**Scheme 1:**
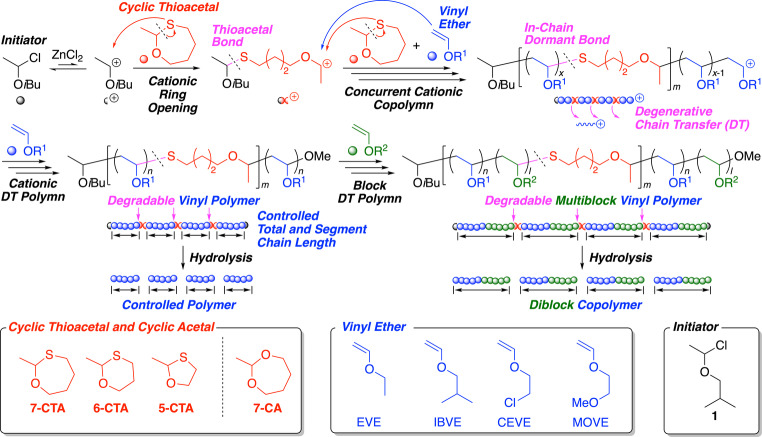
Precision synthesis and degradation of poly(vinyl ether)s and multiblock copolymers with periodically distributed thioacetal bonds in main chains by cationic degenerative chain‐transfer copolymerization of vinyl ethers and cyclic thioacetals.

Considering this background, we are focused on cyclic thioacetals as new comonomers for vinyl monomers as well as precursors for in‐chain dormant species of the resulting vinyl polymers (Scheme 1). In particular, we envision that cyclic thioacetal could cationically copolymerize with vinyl ether via possible ring opening to result in main‐chain thioacetal C−S bonds, which could further serve as in‐chain dormant bonds to enable continuous controlled growth of internal segments via cationic polymerization of vinyl ethers based on the DT mechanism. As a result, poly(vinyl ether)s with periodically distributed thioacetal bonds in the main chains could be obtained. Furthermore, thioacetal bonds can be cleaved by hydrolysis using organic acids and metal salts,[^73^, ^74^, ^75^, ^76^, ^77^] whereas they can survive some postpolymerization modification reactions.^[60]^ This strategy could thus lead to vinyl polymers with precision degradability as well as sufficient stability.

In previous works, cyclic acetals have been cationically copolymerized with vinyl ethers to form degradable poly(vinyl ether)s possessing main‐chain acetal bonds,[^55^, ^56^] whereas the acetal bonds do not serve as dormant species[^59^, ^61^, ^66^] and are thus heterogeneously distributed in the main chains. On the other hand, cyclic trithiocarbonates have been used for radical copolymerization with styrene and acrylate to result in main‐chain trithiocarbonate bonds, which serve as macro‐RAFT agents to result in copolymers containing periodically distributed trithiocarbonate bonds.[^78^, ^79^, ^80^, ^81^] However, trithiocarbonate bonds are labile and can generate products with color and odor. In addition, we previously reported that main‐chain thioacetal bonds of polythioacetals, which are prepared by step‐growth cationic thiol‐ene polymerization of dithiol and divinyl ether, can serve as macro‐chain‐transfer agents for cationic polymerization of vinyl ethers to form copolymers.[^82^, ^83^] However, the total polymer chain lengths cannot be controlled due to the step‐growth mechanism for the formation of polythioacetals in the first step.

Here, we synthesize a series of cyclic thioacetals with 5‐, 6‐, and 7‐membered rings (**5‐CTA**, **6‐CTA**, and **7‐CTA**) and investigate their cationic DT copolymerizations with various vinyl ethers to synthesize poly(vinyl ether)s with periodically distributed thioacetal bonds in the main chains (Scheme 1). The obtained copolymers are degraded into low‐molecular‐weight poly(vinyl ether)s with controlled chain lengths using an organic acid or a metal salt. Furthermore, the one‐pot synthesis of degradable multiblock copolymers, consisting of diblock poly(vinyl ether)s connected by thioacetal bonds, is also reported.

## Results and Discussion

### Synthesis of Cyclic Thioacetals

A series of acetaldehyde‐type 5–7‐membered cyclic thioacetals were synthesized because linear acetaldehyde‐type thioacetals, which are adducts of vinyl ether and thiol, are known as suitable chain‐transfer agents for the cationic DT polymerization of vinyl ethers.^[60]^ The 5‐membered cyclic thioacetal (**5‐CTA**) was synthesized from acetaldehyde dimethyl acetal and 2‐mercaptoethanol, whereas the 6‐membered thioacetal (**6‐CTA**) was prepared from acetaldehyde diethyl acetal 3‐mercapto‐1‐propanol (Figure S1 and S2). The 7‐membered cyclic thioacetal (**7‐CTA**) was synthesized from 4‐(acetylthio)butyl vinyl ether, which was prepared by tosylation of 4‐hydroxybutyl vinyl ether and subsequent thioacetylation. Deprotection of the acetyl group of 4‐(acetylthio)butyl vinyl ether with *n*‐butylamine followed by in situ intramolecular cationic thiol‐ene cyclization using a benzenesulfonic acid as an acid catalyst under dilution conditions led to **7‐CTA** (Figure S3). The obtained cyclic thioacetals could serve as cyclic comonomers for concurrent cationic copolymerization with vinyl ether to generate main‐chain thioacetal bonds, which then function as in‐chain chain‐transfer agents for subsequent cationic DT polymerization of vinyl ether.

### Cationic DT Copolymerization of Ethyl Vinyl Ether and Cyclic Thioacetals

Cationic copolymerization of ethyl vinyl ether (EVE) and a series of cyclic thioacetals was examined using the HCl‐adduct of isobutyl vinyl ether (**1**) as an initiator in conjunction with ZnCl_2_ as a Lewis acid catalyst in the mixture of CH_2_Cl_2_ and Et_2_O (solvent for ZnCl_2_) at −40 °C because the **1**/ZnCl_2_‐initiating system is effective for living cationic polymerization of vinyl ethers.^[84]^ In the first attempt, 10 equivalents of cyclic thioacetals and 200 equivalents of EVE to **1** were used. Thus, the feed ratio of EVE to cyclic thioacetals was 20 ([EVE]_0_/[**7‐CTA**]_0_=20/1).

**Figure 1:**
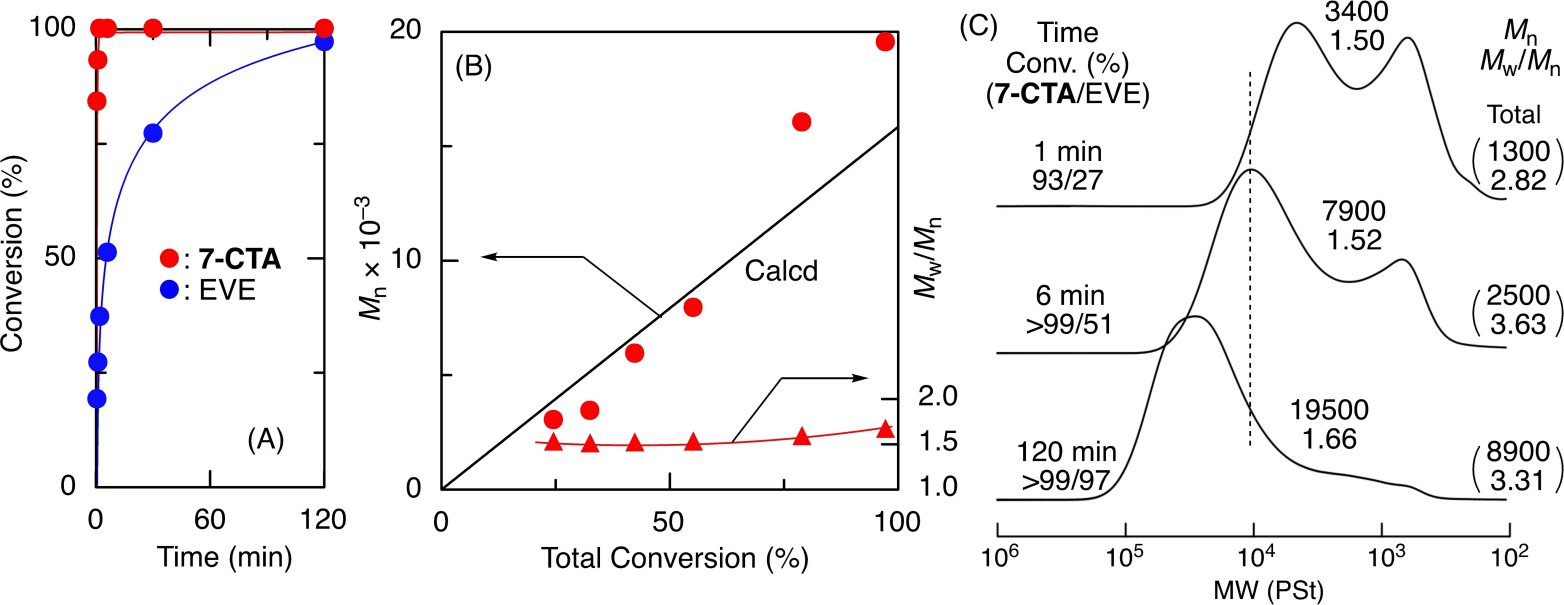
Time‐conversion curve (A), *M*_n_ values (B), and SEC curves (C) of the polymers obtained in controllled cationic copolymerization of EVE and **7‐CTA**: [EVE]_0_/[**7‐CTA**]_0_/[**1**]_0_/[ZnCl_2_]_0_=4000/200/20/4.0 mM in CH_2_Cl_2_/*n*‐hexane/Et_2_O (20/10/10) at −40 °C.

Whereas **5‐CTA** and **6‐CTA** were not consumed at all to result in homopolymer of EVE (Figure S4–S6) most probably due to the stable 5‐ and 6‐membered ring, **7‐CTA** was consumed very rapidly (≥99 % in 6 min) along with a slower (27 % in 6 min) but nearly complete subsequent consumption (97 % in 120 min) of EVE (Figure 1A). The consumption of **7‐CTA** was much faster than that of a similar 7‐membered cyclic acetal (**7‐CA**) (Figure S4), which resulted in statistical copolymers of EVE (Figure S7 and S8).[^55^, ^56^] Thus, **7‐CTA** has a high reactivity in cationic polymerization.

Although size‐exclusion chromatography (SEC) curves of the products obtained with **7‐CTA** and EVE were bimodal in the initial stages of copolymerization, they became almost unimodal and shifted to the high‐molecular‐weight regions along with consumption of EVE (Figure 1C). The number‐average molecular weights (*M*_n_) of the main products with a higher‐molecular‐weight peak increased with monomer conversion, consistent with the calculated values assuming one molecule of **1** generated one copolymer chain, and finally reached 19 500 (Figure 1B).

**Figure 2:**
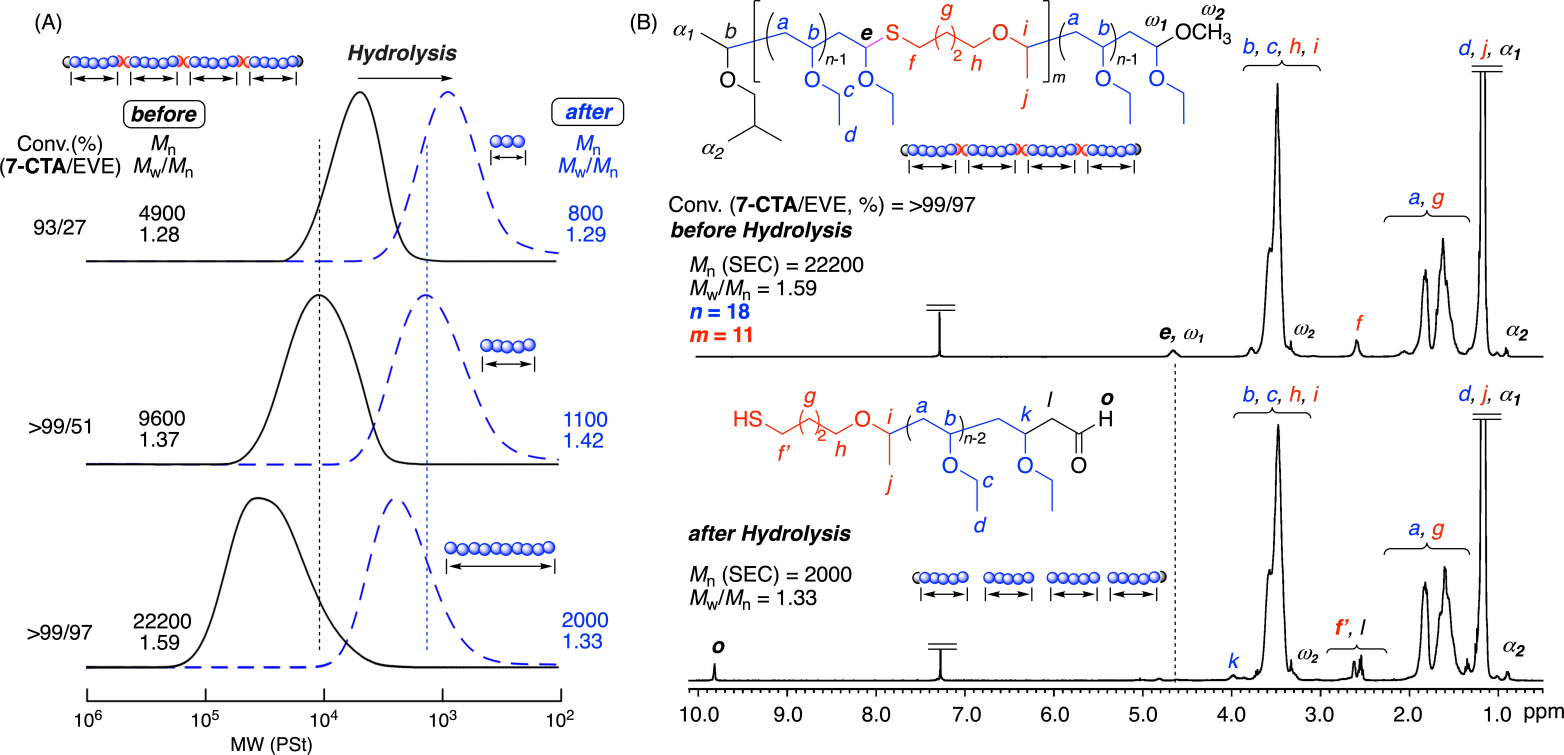
SEC curves (A) and ^1^H NMR spectra (CDCl_3_, 55 °C) (B) of the polymers obtained before and after degradation reaction using an AgNO_3_ solution: [thioacetal unit]_0_/[AgNO_3_]_0_=5.0/50 mM in THF/H_2_O at 20 °C.

The main products for each sample obtained at different EVE conversions were separated from the minor products with lower molecular weights using preparative SEC and were analyzed by ^1^H NMR spectroscopy (Figure 2B and S9). In all the spectra, characteristic peaks assignable to the methine proton of thioacetal (*e*) and methylene protons adjacent to sulfur atom (*f*) in the ring‐opened structures were observed at 4.7 and 2.6 ppm, respectively, in addition to the protons of the poly(EVE) units. Thus, **7‐CTA** underwent ring‐opening polymerization and was copolymerized with EVE to be incorporated into the main chains.

Furthermore, the methyl proton (α_2_) of the isobutyl group at the α‐end derived from the initiator (**1**) and the methoxy proton (ω_2_) at the ω‐end originating from methanol as a quencher were observed. The terminal acetal methine proton (ω_1_) should overlap with the thioacetal methine proton (*e*). Average numbers (*m*) of the incorporated **7‐CTA** units were calculated from the integral ratios of *f* to α_2_ and were close to 10, regardless of the EVE consumption. The *m* values were in good agreement with the theoretical values calculated from the feed ratio of **7‐CTA** to **1** ([**7‐CTA**]_0_/[**1**]_0_×conversion of **7‐CTA**), indicating that **7‐CTA** was quantitatively copolymerized and incorporated into the main chains. In particular, the *m* value for the final products was 11 (Figure 2B). On the other hand, the number‐average degrees (*n*) of poly(EVE) segments, which were calculated from the integral ratios of EVE units to the sum of in‐chain thioacetal and terminal acetal bonds, increased with consumption of EVE and finally reached 18 (Figure 2B). This value was close to the theoretical value ([EVE]_0_/([**7‐CTA**]_0_+[**1**]_0_)=18.2), assuming that all **7‐CTA** and **1** molecules served as reversible‐chain transfer agents and initiators, respectively. These results indicate that the copolymer chain of the final products was composed of 11 (=*m*) thioacetal bonds and 12 (=*m*+1) poly(EVE) segments with 18 (=*n*) EVE units on average and that these values were close to what we targeted (*m*=10, *n*=18.2).

As shown in Figure 1C, broadening of the MWDs of the higher‐molecular‐weight peak was observed. This result suggests a concurrently occurring shuffling reaction of poly(EVE) segments among polymer chains via the cationic DT mechanism (see below). Similar broadening of MWDs was reported in scrambling reactions among the polymer segments connected by dynamic covalent bonds such as alkoxyamine.^[85]^

The minor products with lower molecular weights obtained in the initial stages of copolymerization were fractionated by preparative SEC and analyzed by ^1^H NMR. The *M*_n_(NMR, α‐end) value calculated from the integral ratio of EVE units to the α‐end group (α_2_) was 7000, which was much higher than that measured by SEC (*M*_n_(SEC)=700) as well as that calculated from the thioacetal protons (*M*_n_(NMR, thioacetal)=450) (Figure S10). These results indicate that the minor products were mainly cyclic oligomers with thioacetal bonds, which can also participate in the cationic polymerization of EVE via the DT mechanism in the later stages to be incorporated into high‐molecular‐weight polymers (see below).

### Degradation of Copolymers

Degradation of the copolymers was investigated via hydrolysis of the main‐chain thioacetal bonds using metal salts or organic acids. To prove the degradability more clearly, AgNO_3_ was used as the first choice in the mixture of THF and H_2_O at 20 °C because this salt is known to cleave thioacetal bonds efficiently.^[76]^ The main products with a higher‐molecular‐weight peak were similarly fractionated by preparative SEC and were subjected to this reaction. In the ^1^H NMR spectrum of the products obtained after 3 h, the peaks attributed to the thioacetal (*e*) groups disappeared (Figure 2B). In addition, a new peak was observed at 9.8 ppm and was attributed to the aldehyde chain end, which was generated via cleavage of thioacetal bonds.

Figure 2A shows the SEC curves of the copolymers obtained at different EVE conversions before and after treatment with the AgNO_3_ solution. All SEC curves shifted to the low‐molecular‐weight regions, maintaining the unimodal and narrow MWDs. In addition, the *M*_n_ values of the degraded products were in good agreement with the calculated values, assuming that the length of each poly(EVE) segment was eventually controlled by the feed ratio of EVE and **7‐CTA** (Figure S11). Thus, precision degradation of the copolymers occurred, resulting in low‐molecular‐weight polymers with arbitrarily controlled molecular weights.

Furthermore, the degradation of the copolymers using *p*‐toluenesulfonic acid (PTSA) as a common organic catalyst was examined under similar conditions. Although the reaction was slower than that with AgNO_3_, the thioacetal bonds were degraded to result in low‐molecular‐weight polymers with unimodal and narrow MWDs in 2–4 weeks (Figures S12 and S13).

As a control experiment, the degradation of copolymers obtained from EVE and cyclic acetal (**7‐CA**) was similarly conducted using PTSA under the same conditions. The acetal bonds were also completely degraded in 24 h, resulting in products with a terminal aldehyde group (Figure S8B). The SEC curve shifted to the low‐molecular‐weight regions, with broadening and tailing (Figure S4). The MWD of the degraded polymers was broader than that obtained for **7‐CTA**. These results indicate that the acetal bonds were introduced heterogeneously in the poly(EVE) main chains via cationic copolymerization, where **7‐CA** has a low monomer reactivity ratio. Another significant difference compared to **7‐CTA** is that the introduced in‐chain acetal bonds were hardly activated during the cationic polymerization of EVE because an acetal is not a suitable reversible chain‐transfer agent.[^59^, ^61^] Furthermore, the acetal linkage was not susceptible to AgNO_3_, which will enable selective degradation of acetal or thioacetal bond.

Thus, **7‐CTA** is highly effective at producing poly(EVE) with periodically distributed degradable thioacetal bonds in the main chains via the one‐shot concurrent cationic copolymerization of **7‐CTA** and EVE via the DT mechanism.

### Variable Total and Segment Chain Lengths for Precision Synthesis and Degradation of Copolymers

This cationic DT copolymerization could control not only the total lengths of the copolymers by the feed ratio of the total monomers (EVE and **7‐CTA**) to the initiator (**1**) but also the segment lengths of poly(EVE) sandwiched between the thioacetal bonds by the feed ratio of EVE to **7‐CTA**. The feed ratios were thus systematically changed to achieve precision synthesis and degradation of the copolymers.

**Figure 3:**
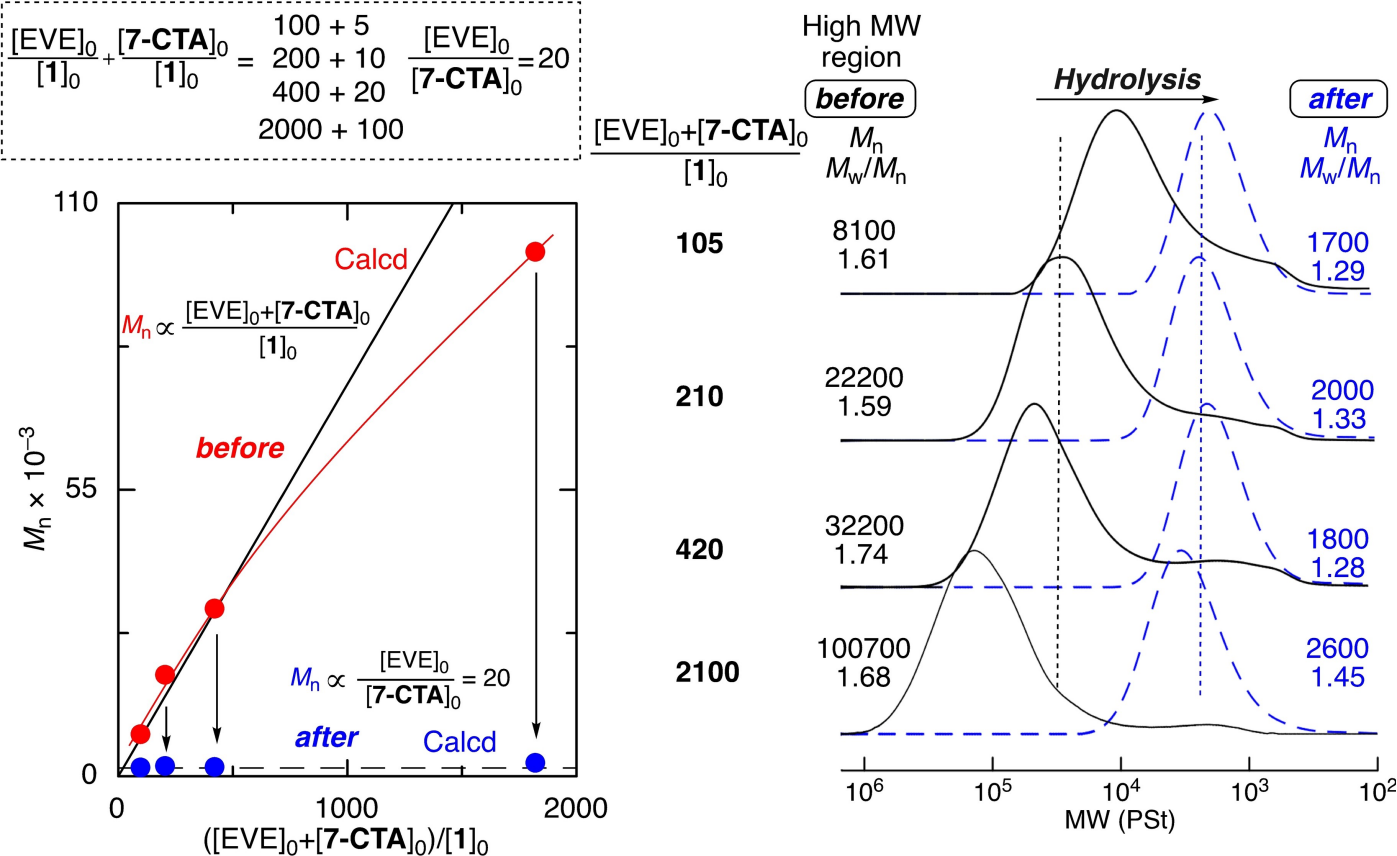
Effect of varying ([EVE]_0_+[**7‐CTA**]_0_)/[**1**]_0_ with constant [EVE]_0_/[**7‐CTA**]_0_ on the polymers obtained in controlled cationic copolymerizations of EVE and **7‐CTA** before and after hydrolysis: [EVE]_0_/[**7‐CTA**]_0_/[**1**]_0_/[ZnCl_2_]_0_=4000/200/10, 20, 40/4.0 or 6000/300/3.0/6.0 mM in CH_2_Cl_2_/*n*‐hexane/Et_2_O (20/10/10) at −40 °C. [thioacetal unit]_0_/[AgNO_3_]_0_=5.0/50 mM in THF/H_2_O at 20 °C

To vary the total chain lengths but keep the segment lengths constant, the feed ratio of (EVE+**7‐CTA**) to **1** was varied from (100+5) to (2000+100), while that of EVE to **7‐CTA** was kept constant at 20. In all cases, **7‐CTA** was consumed much faster than EVE (Figure S14) to result in the copolymers (Figure S15). The SEC curves of the products shifted to the high‐molecular‐weight regions as the feed ratio of the monomers to the initiator was increased (Figure 3). Moreover, the *M*_n_ of the main products increased almost linearly and agreed well with the theoretical value. At the highest feed ratio of (2000+100), high‐molecular‐weight polymers with *M*_n_ over 100 000 were formed, although this was slightly lower than the theoretical value.

Then, the degradation of a series of polymers was examined using an AgNO_3_ solution as before. In all cases, the SEC curves significantly shifted to almost the same low‐molecular‐weight regions and showed unimodal and narrow MWDs (Figure 3).

The *M*_n_ of all the products was approximately 2 000, which was close to the theoretical values determined by the constant feed ratio of EVE to **7‐CTA**, regardless of the differences in the initial molecular weights ranging from 8 000 to 100 000. A series of degradable polymers, which have different total molecular weights but can be degraded into the same molecular weights, were thus prepared.

**Figure 4:**
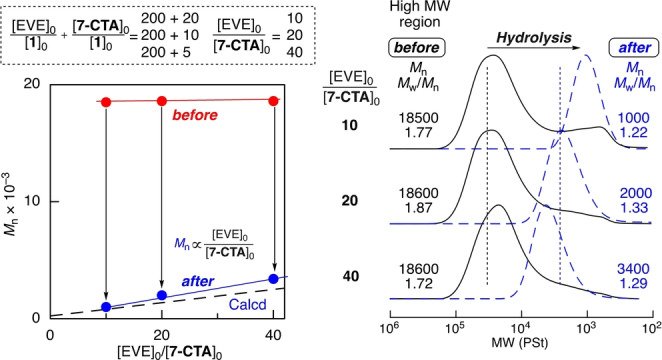
Effect of varying [EVE]_0_/[**7‐CTA**]_0_ with constant [EVE]_0_/[**1**]_0_ on the polymers obtained in controlled cationic copolymerizations of EVE and **7‐CTA** before and after hydrolysis: [EVE]_0_/[**7‐CTA**]_0_/[**1**]_0_/[ZnCl_2_]_0_=4000/100, 200, 400/20/4.0 mM in CH_2_Cl_2_/*n*‐hexane/Et_2_O (20/10/10) at −40 °C. [thioacetal unit]_0_/[AgNO_3_]_0_=5.0/50 mM in THF/H_2_O at 20 °C.

For another series of copolymers having almost the same total molecular weights but different segment lengths, the feed ratio of EVE to **7‐CTA** was varied from 10 to 20 and 40, while that of EVE to **1** was kept constant at 200. **7‐CTA** was similarly consumed quickly, followed by quantitative consumption of EVE (Figure S16). The *M*_n_ values of the products were almost the same (Figure 4 and Figure S17). The ^1^H NMR analysis revealed that the number‐average degree (*n*) of poly(EVE) segments increased from 7.9 to 18 and 27 as the feed ratio of EVE to **7‐CTA** was increased (Figure S17). These results support the formation of copolymers with almost the same molecular weights but different segment chain lengths.

Degradation of these polymers was similarly conducted. The SEC curves shifted to the low‐molecular‐weight regions at different positions (Figure 4). In particular, the *M*_n_ values of the degraded products were close to the values calculated from the feed ratio of EVE to **7‐CTA** and increased in direct proportion to the feed ratio. Thus, another series of degradable polymers, which have the same total molecular weights but can be degraded into different molecular weights, were synthesized.

These results indicate that precision synthesis and degradation of poly(EVE) is achievable by simply changing the feed ratios of EVE, **7‐CTA**, and **1** in the cationic DT copolymerizations.

### Polymerization Mechanism

According to the kinetics shown in Figure 1, most of **7‐CTA** is consumed in the initial stages of the copolymerization with a small portion of large amount of EVE ([EVE]_0_/[**7‐CTA**]_0_=20/1) to produce the co‐oligomers having thioacetal bonds in the main chains (Scheme 1). Subsequently, the in‐chain thioacetal bonds are reactivated via DT reaction to generate the cationic propagating species from the in‐chain dormant species, into which the remaining large amount of EVE is schematically inserted to increase lengths of the poly(EVE) segment and thereby the total molecular weights. The lengths of poly(EVE) segments as well as whole chains are controlled via the reversible (de)activation process.

**Scheme 2:**
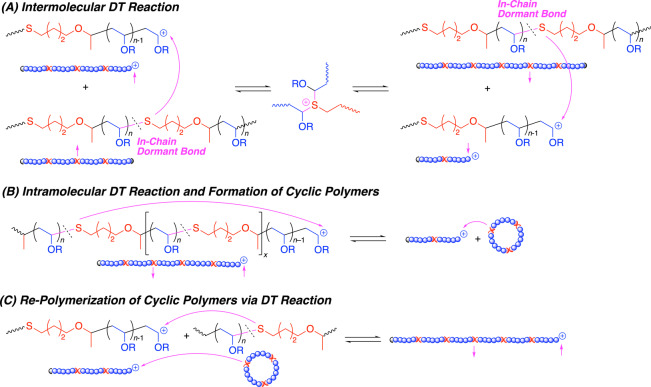
Various DT reactions affecting molecular weights and structures of resulting polymers.

However, an intermolecular DT reaction (Scheme 2A) induces shuffling of the polymer segments among the polymer chains, which causes broadening of MWDs of the whole polymer chains, although the total *M*_n_ values are controlled by the feed ratio of monomers to initiator. In addition, a DT reaction could also occur intramolecularly (Scheme 2B) to result in cyclic oligomers, which causes bimodal peaks and tailing in the SEC curves. The formation of cyclic oligomers was supported by ^1^H NMR analysis of the low‐molecular‐weight‐peak products (Figure S10) as mentioned above. However, these cyclic polymers are not inert and can be re‐incorporated into other linear polymer chains via another DT reaction (Scheme 2C), which is a back reaction of the intramolecular DT reaction (Scheme 2B). Actually, the peak intensity of low‐molecular‐weight products decreased as EVE was consumed.

Thus, the cationic DT polymerization allows control of lengths of the internal poly(EVE) segments and the total polymer chains although the intermolecular and intramolecular DT reactions cause broadening of MWDs of the whole polymer chains and low‐molecular‐weight cyclic products particularly in the initial stage of the polymerizations. These are characteristic features prone to the DT polymerizations involving in‐chain dormant species to produce vinyl polymers possessing carbon‐heteroatom bonds in the main chains.

### Cationic DT Copolymerization of Various Vinyl Ethers with 7‐CTA

To confirm the versatility of the system consisting of **1**, **7‐CTA** and ZnCl_2_, various vinyl ethers, such as isobutyl (IBVE), 2‐chloroethyl (CEVE), and 2‐methoxyethyl vinyl ether (MOVE), were used under the same conditions as for EVE, as shown in Figure 1. Here, IBVE is one of the most common alkyl vinyl ethers used for living cationic polymerization studies.^[86]^ While CEVE can be used for postpolymerization modification reactions, MOVE results in hydrophilic polymers.

**Figure 5:**
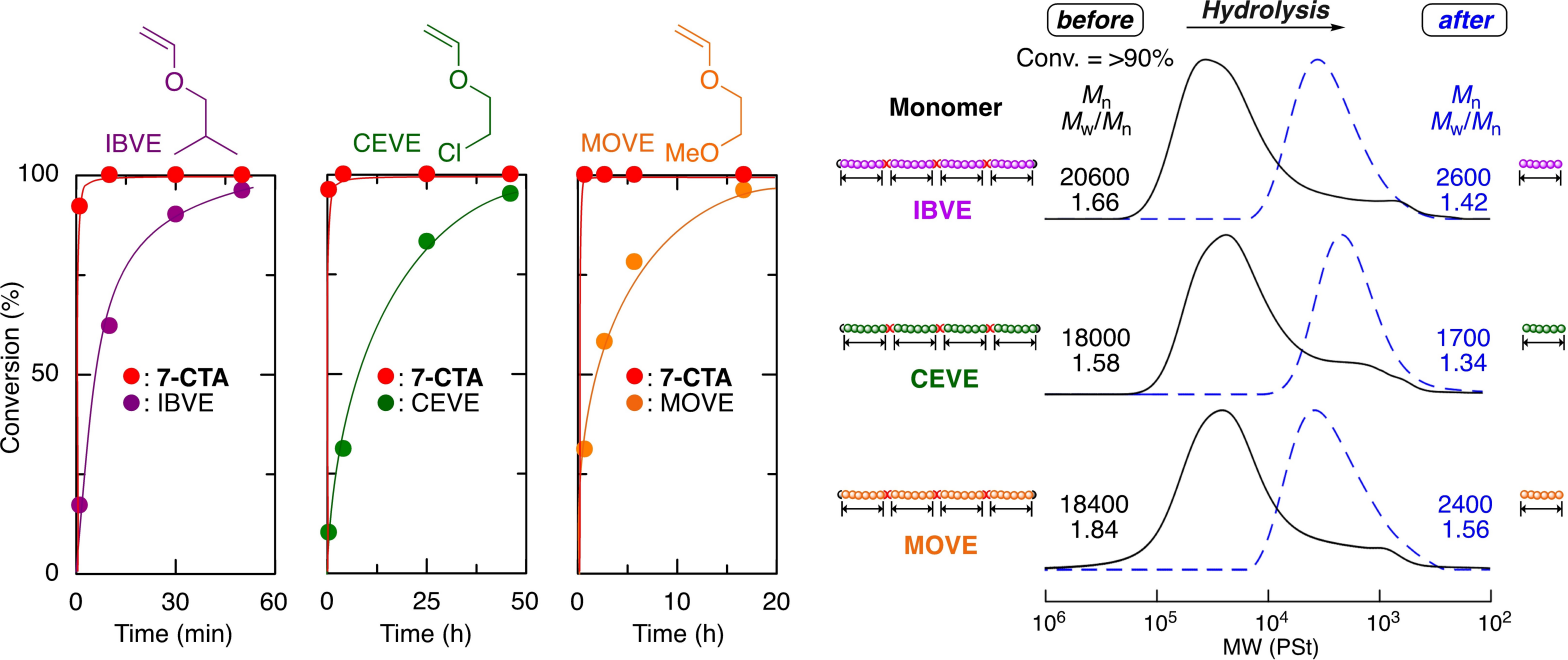
Controlled cationic copolymerization of various vinyl ethers with **7‐CTA** before and after hydrolysis: [VE]_0_/[**7‐CTA**]_0_/[**1**]_0_/[ZnCl_2_]_0_=4000/200/20/4.0 mM in CH_2_Cl_2_/*n*‐hexane/Et_2_O (20/10/10) at −40 °C. [thioacetal unit]_0_/[AgNO_3_]_0_=5.0/50 mM in THF/H_2_O at 20 °C.

In all cases, **7‐CTA** was consumed first, followed by quantitative consumption of vinyl ethers (Figure 5). The *M*_n_ values of the products all increased in direct proportion to monomer conversions, consistent with the theoretical values, and finally resulted in polymers with high and controlled molecular weights (Figure S18). In the ^1^H NMR spectra of the polymers obtained at the highest conversion (Figures S19–S21), peaks assignable to each vinyl ether unit and **7‐CTA** were observed. In addition, the average numbers (*m*) of the incorporated **7‐CTA** units calculated from the integral ratios were 10–11, which were all close to the theoretical value, i.e., 10. The number‐average degrees (*n*) of polymerization of vinyl ethers were 15–18, which were slightly lower than the theoretical values. These results indicate that various vinyl ethers can be similarly copolymerized with **7‐CTA** in a controlled fashion to result in copolymers consisting of poly(vinyl ether) segments linked by main‐chain thioacetal units.

The degradation of these copolymers was also investigated using an AgNO_3_ solution under the same conditions as described above. All copolymers were converted into low‐molecular‐weight polymers with narrow MWDs and *M*_n_ values close to the theoretical values determined by the feed ratio of vinyl ethers to **7‐CTA** (Figure 5), again indicating precision degradation. Thus, this system is applicable to various vinyl ethers and enables the precision synthesis of hydrophobic, hydrophilic, or functional poly(vinyl ether) with degradability.

### Synthesis of Degradable Multiblock Copolymers and Degradation into Diblock Copolymers

**Figure 6:**
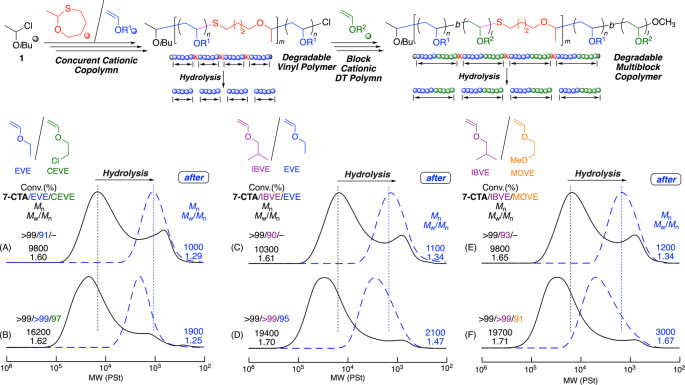
One‐pot synthesis of degradable multiblock copolymers linked by thioacetal bonds and hydrolysis into the diblock copolymers: [VE]_0_/[VE]_add_/[**7‐CTA**]_0_/[**1**]_0_/[ZnCl_2_]_0_=2000/2000/200/20/4.0 mM in CH_2_Cl_2_/*n*‐hexane/Et_2_O (20/10/10) at −40 °C. [thioacetal unit]_0_/[AgNO_3_]_0_=5.0/50 mM in THF/H_2_O at 20 °C.

Taking advantage of the in‐chain dormant thioacetal bonds, we investigated the one‐pot synthesis of degradable multiblock copolymers composed of diblock copolymer segments connected by thioacetal bonds via the sequential addition of the second monomer to the cationic DT copolymerization of the first monomer and **7‐CTA**. Here, for the first example, for the cationic DT copolymerization of EVE and **7‐CTA**, an equimolar amount of CEVE was added to EVE when EVE was almost consumed (Figure S22). The added CEVE was also consumed nearly quantitatively, resulting in a shift of the main peak of the SEC curves to the high‐molecular‐weight region, indicating the formation of block copolymers via the one‐pot sequential monomer addition (Figure 6A and 6B).

For the other examples, block copolymerizations of not only IBVE and EVE but also IBVE and MOVE were also examined. In both cases, the second monomer (EVE or MOVE) was added to the cationic DT copolymerization IBVE and **7‐CTA** in a similar manner (Figures S23 and S24). After the addition, the SEC curves similarly shifted (Figure 6C–6F), again indicating the formation of a series of block copolymers consisting of hydrophobic, hydrophilic, and functional units.

All these block copolymers were similarly subjected to an AgNO_3_ solution, resulting in degraded polymers with low molecular weights and narrow MWDs (blue dotted lines in Figure 6B, 6D, and 6F). In addition, these SEC curves were located in the high‐molecular‐weight regions compared with those obtained from the prepolymers (blue dotted lines in Figure 6A, 6C, and 6E, respectively). These results suggest that the block copolymers obtained by the sequential monomer addition were multiblock copolymers consisting of diblock poly(vinyl ether) segments linked by thioacetal bonds and that the degraded products were the diblock copolymers.

**Figure 7:**
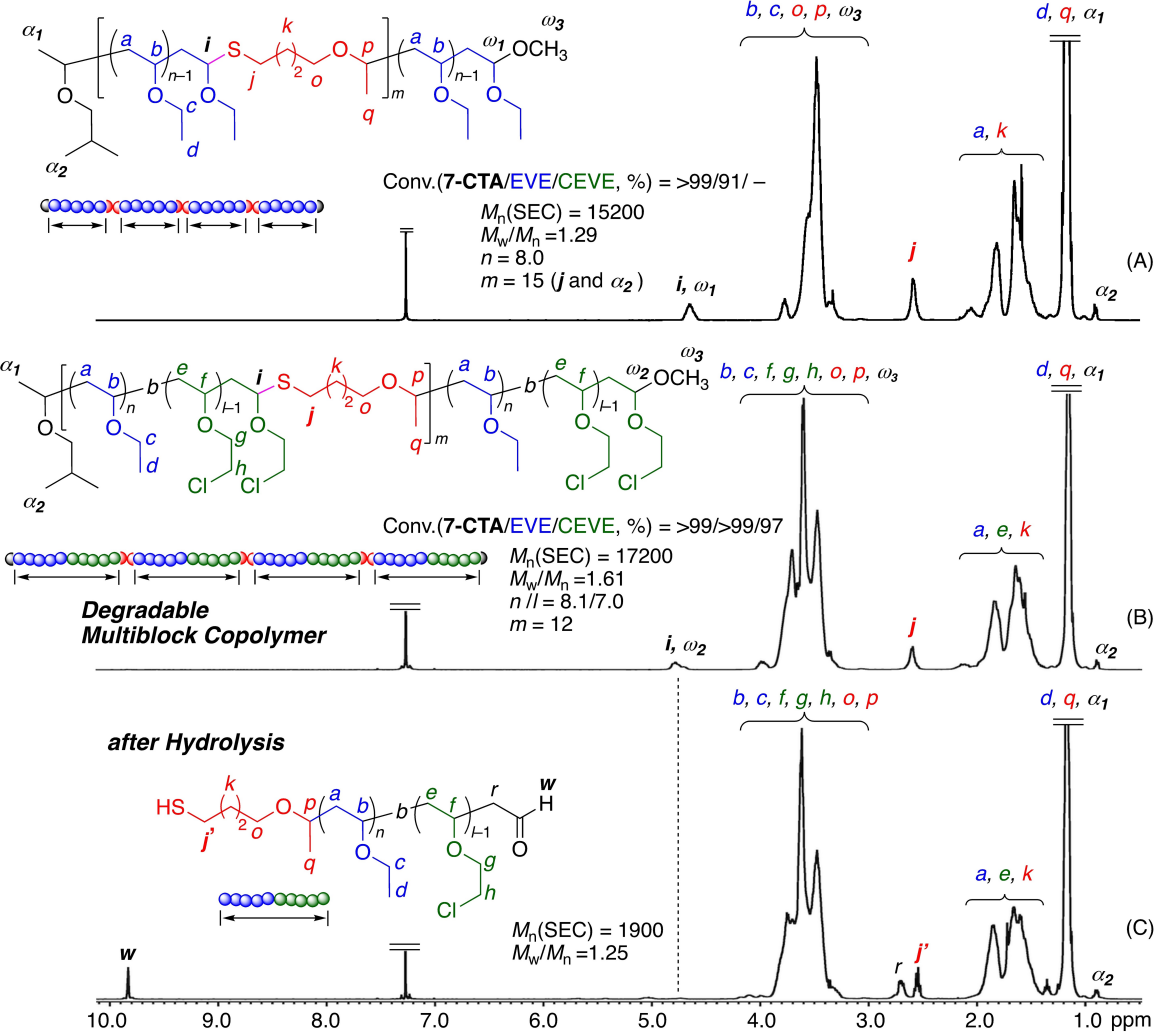
^1^H NMR spectra (CDCl_3_, 55 °C) of degradable poly(EVE) (A), multiblock poly(EVE‐CEVE) (B), and the polymers obtained after hydrolysis of multiblock poly(EVE‐CEVE) (C).

Moreover, a series of ^1^H NMR analyses also support the formation of degradable multiblock copolymers linked by thioacetal linkages. Figure 7 shows the ^1^H NMR spectra of the obtained polymers at high‐molecular‐weight regions after purification by preparative SEC. For the combination of EVE and CEVE, the spectrum obtained after the addition of CEVE (Figure 7B) clearly indicates additional signals of CEVE units (Figure 7A). The number‐average degrees of polymerization of EVE (*n*) and CEVE (*l*), calculated from the integral ratio of each peak, were 8.1 and 7.0, respectively. In addition, the average number (*m*) of thioacetal groups in the main chain was 12. These results indicate the formation of the multiblock copolymer, in which 13 (=*m*+1) diblock copolymers of EVE and CEVE, both with approximately 8 monomer units on average, were connected with thioacetal groups.

Furthermore, the spectrum after degradation showed the complete disappearance of thioacetal methine protons (*i*) at 4.7 ppm and the appearance of new peaks assignable to the terminal aldehyde (*w*) and the adjacent methylene (*r*) protons, while the peaks of EVE and CEVE units remained almost unchanged (Figure 7C). Thus, degradable multiblock copolymers composed of poly(IBVE‐*b*‐CEVE) diblock copolymer segments connected with thioacetal groups were formed and efficiently degraded into diblock copolymers. Similar results were also obtained for the above prepared poly(IBVE‐*b*‐EVE) (Figure S25) and poly(IBVE‐*b*‐MOVE) (Figure S26).

Therefore, precision one‐pot synthesis of degradable multiblock copolymers is achievable by cationic DT copolymerization of cyclic thioacetal and vinyl ether via sequential monomer additions.

### Sequential Cationic Copolymerization of Cyclic Thioacetals and Vinyl Ethers for Mechanistic Study

While mixtures of vinyl ethers and cyclic thioacetals have thus far been copolymerized for the synthesis of degradable vinyl polymers, the kinetics of the copolymerizations could lead to the hypothesis that similar copolymers may be synthesized by sequential cationic copolymerization, i.e., cationic ring‐opening homopolymerization of cyclic thioacetals and subsequent cationic DT polymerization of vinyl ethers inserting into preformed in‐chain thioacetal dormant bonds. Finally, to clarify the mechanism for the formation of copolymers of vinyl ethers and cyclic thioacetals, the sequential cationic copolymerization of **7‐CTA** and EVE was examined.

The cationic ring‐opening homopolymerization of **7‐CTA** was first carried out using **1**/ZnCl_2_ at −40 °C in CH_2_Cl_2_ (Figure S27). When **7‐CTA** was nearly consumed (conversion=91 %), EVE was added. The added EVE was also consumed quantitatively by the subsequent polymerization. After consumption of EVE, the multimodal SEC curve of preformed poly(**7‐CTA**) was shifted to the high‐molecular‐weight region, resulting in the unimodal SEC curve (Figure S28), which was similar to that obtained in the one‐shot cationic copolymerization of **7‐CTA** and EVE (Figure 1). However, treatment of the products with an AgNO_3_ solution resulted in only a slight decrease in *M*_n_ to 7600, where the decrease was not significant in comparison to that for the products obtained by the one‐shot copolymerization.

^1^H NMR analysis revealed that the products obtained in the sequential addition experiments before and after EVE addition contained not only thioacetal bonds but also dithioacetal and acetal bonds in the main chain (Figure S29). In particular, the ratio of thioacetal/dithioacetal/acetal in the main chains of the preformed poly(**7‐CTA**) was 14/43/43, suggesting that thioacetal was converted into acetal and dithioacetal during the ring‐opening cationic homopolymerization of **7‐CTA** in the first stage. A similar formation of dithioacetal and acetal linkages by cationic ring‐opening homopolymerization of a formaldehyde‐type 5‐membered cyclic thioacetal has been reported.[^87^, ^88^]

**Scheme 3:**
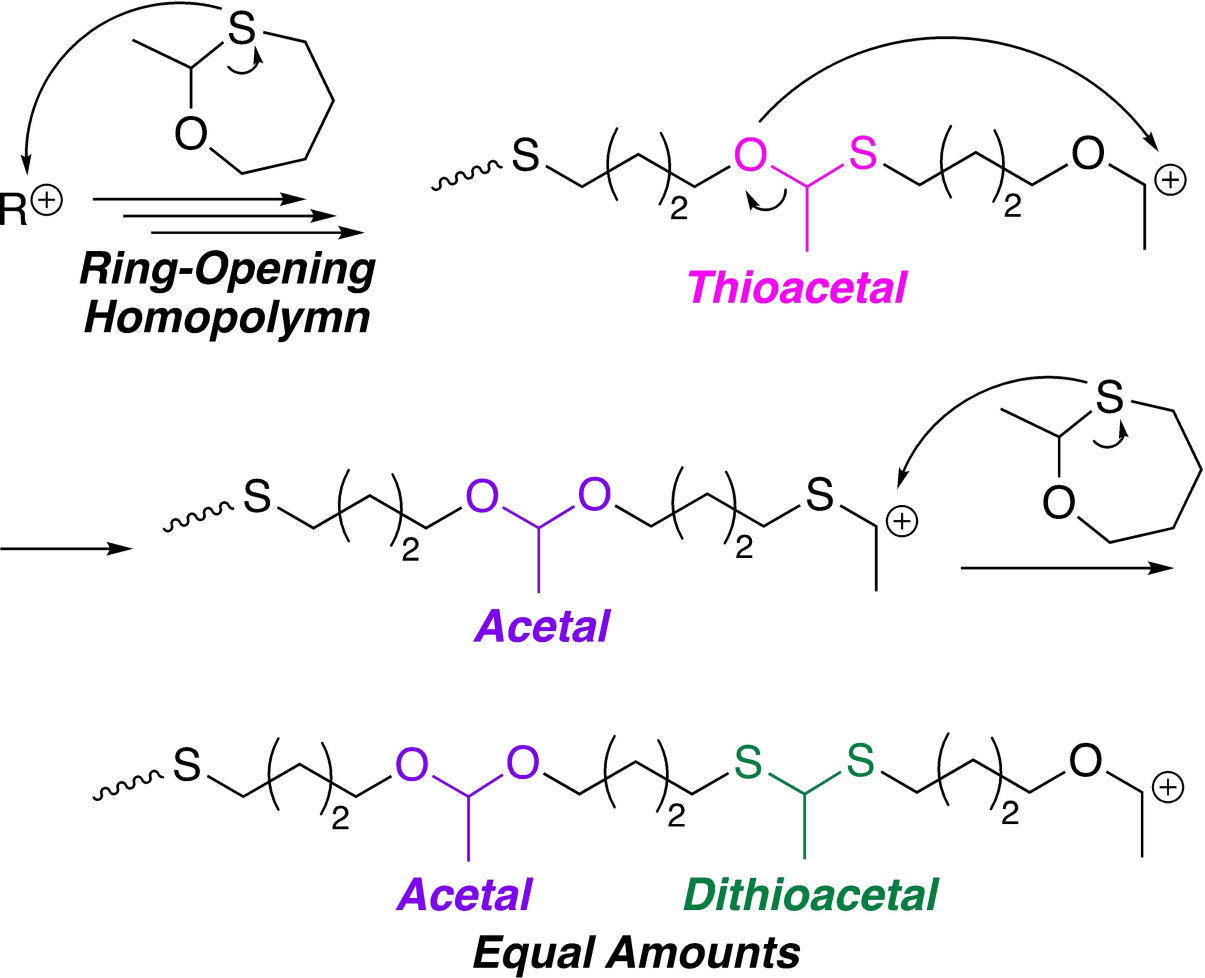
Formation of acetal and dithioacetal bonds during cationic ring‐opening of homopolymerization of 7‐CTA.

Then, the cationic ring‐opening homopolymerization was examined in more detail (Figures S30–S32). The obtained products again showed acetal and dithioacetal bonds. Furthermore, the contents of acetal and dithioacetal bonds were almost the same and increased as the polymerization proceeded. Along with this change, the ratio of thioacetal decreased. These results indicate that back‐biting or chain‐transfer reactions to oxygen atoms of the main‐chain thioacetal bonds occurred during the homopolymerization of **7‐CTA** to generate the acetal and dithioacetal bonds (Scheme 3). Since acetal and dithoacetal bonds were not observed in the concurrent copolymerizations, the exchange reaction was most likely caused by an intramolecular back‐biting reaction to the oxygen atom of the penultimate thioacetal monomer unit.

A closer look at the one‐shot copolymerization revealed that a small amount of EVE was consumed with **7‐CTA** in the initial stages (e.g., Figure 1), suggesting that a small amount of EVE might be required to suppress the formation of dithioacetal and acetal bonds. To investigate this possibility, cationic copolymerization of a small amount of EVE and **7‐CTA** at a feed ratio of 2.5 to 1 was first conducted. After nearly complete consumption of the initial feed, EVE was subsequently added so that the total feed ratio of EVE to **7‐CTA** was 200 to 1 (Figure S33). The products obtained both before and after monomer addition did not contain any dithioacetal linkages in the main chains (Figure S34). In addition, the average number (*m*) of thioacetal groups in the main chain was 10, in good agreement with the theoretical value. These results indicate that the formation of dithioacetal and acetal bonds was suppressed by EVE, which can concurrently copolymerize with **7‐CTA**. Finally, treatment of the products with an AgNO_3_ solution led to a clear shift of the SEC curve from *M*_n_=18 000 to 2000 and a narrow MWD (Figure S35), which was quite similar to that obtained by the one‐shot cationic copolymerization.

Thus, the presence of vinyl ethers is requisite for precision formation of thioacetal bonds in the main chain of the resulting polymers during the cationic polymerization of 7‐membered cyclic thioacetals, and concurrent cationic copolymerization enables precision synthesis of degradable vinyl polymers.

## Conclusion

Precision synthesis of degradable poly(vinyl ether)s containing periodic thioacetal bonds in the main chains was achieved by concurrent cationic copolymerization of vinyl ethers and 7‐membered cyclic thioacetal. The polymers formed via ring‐opening cationic copolymerization of cyclic thioacetals and a small amount of vinyl ethers followed by controlled cationic polymerization of remaining vinyl ethers via the DT mechanism to main‐chain thioacetal bonds. The obtained polymers were degraded into low‐molecular‐weight poly(vinyl ether)s with narrow molecular weight distributions by hydrolysis using an aqueous solution of AgNO_3_ or *p*‐toluenesulfonic acid. The overall and segment molecular weights were precisely controlled by varying the feed ratios of total monomers to the initiator and vinyl ethers to cyclic thioacetal, respectively. In addition, various vinyl ethers were used to generate degradable poly(vinyl ether)s with hydrophobic, hydrophilic, and functional pendants and thioacetal bonds in the main chains. Furthermore, one‐pot synthesis of degradable multiblock copolymers and precision degradation to diblock poly(vinyl ethers)s were achievable. We hope that this precision synthesis and degradation of vinyl polymers, which is based on concurrent ring‐opening and addition polymerization via the DT mechanism, will contribute to the development of novel degradable polymer materials and solutions to environmental issues caused by waste plastics.

## Conflict of interest

The authors declare no conflict of interest.

1

## Supporting information

As a service to our authors and readers, this journal provides supporting information supplied by the authors. Such materials are peer reviewed and may be re‐organized for online delivery, but are not copy‐edited or typeset. Technical support issues arising from supporting information (other than missing files) should be addressed to the authors.

Supporting Information

## Data Availability

Research data are not shared.
