# Supplementary material for: Synthesis and Degradation of Vinyl Polymers with Evenly Distributed Thioacetal Bonds in Main Chains: Cationic DT Copolymerization of Vinyl Ethers and Cyclic Thioacetals
Source: Angew Chem Int Ed Engl. 2022 Dec 7;62(4):e202215021. doi: 10.1002/anie.202215021 (PMC10107285; doi:10.1002/anie.202215021)
Supplement: Supplementary file 1 — Supporting Information [file ANIE-62-0-s001.pdf]

Angewandte Chemie
Eine Zeitschrift der Gesellschaft Deutscher Chemiker

Supporting Information

Synthesis and Degradation of Vinyl Polymers with Evenly Distributed Thioacetal Bonds in Main Chains: Cationic DT Copolymerization of Vinyl Ethers and Cyclic Thioacetals

M. Uchiyama, Y. Murakami, K. Satoh, M. Kamigaito**

Supporting Information

Contents:

Experimental Section ······ S3
Figure S1 ······ S7
Figure S2 ······ S7
Figure S3 ······ S8
Figure S4 ······ S8
Figure S5 ······ S9
Figure S6 ······ S9
Figure S7 ······ S10
Figure S8 ······ S10
Figure S9 ······ S11
Figure S10 ······ S11
Figure S11 ······ S12
Figure S12 ······ S12
Figure S13 ······ S13
Figure S14 ······ S13
Figure S15 ······ S14
Figure S16 ······ S14
Figure S17 ······ S15
Figure S18 ······ S15

Figure S19 ···························· S16
Figure S20 ···························· S16
Figure S21 ···························· S17
Figure S22 ···························· S17
Figure S23 ···························· S18
Figure S24 ···························· S18
Figure S25 ···························· S19
Figure S26 ···························· S20
Figure S27 ···························· S21
Figure S28 ···························· S21
Figure S29 ···························· S22
Figure S30 ···························· S22
Figure S31 ···························· S23
Figure S32 ···························· S23
Figure S33 ···························· S24
Figure S34 ···························· S24
Figure S35 ···························· S25

Experimental Section

Materials

Ethyl vinyl ether (EVE) (TCI, >98.0%), isobutyl vinyl ether (IBVE) (TCI, >99.0%), 2-chloroethyl vinyl ether (CEVE) (TCI, >97.0%), and 1,2-dichlorobenzene (TCI, >99.0%) were distilled over calcium hydride under reduced pressure before use. 4-Hydroxybutyl vinyl ether (TCI, >97.0%), *p*-toluenesulfonyl chloride (TCI, >99.0%), potassium thioacetate (TCI, >97.0%), butylamine (TCI, >99.0%), benzenesulfonic acid anhydrous (TCI, >98.0%), *p*-toluenesulfonic acid monohydrate (TCI, >98.0%), 2-mercaptoethanol (TCI, >98.0%), 3-mercapto-1-propanol (TCI, >97.0%), acetaldehyde dimethyl acetal (TCI, >98.0%), acetaldehyde diethyl acetal (TCI, >98.0%), triethylamine (TCI, >99.0%) and $ZnCl_2$ (Aldrich; 1.0 mM solution in diethyl ether) were used as received. 2-Methyl-1,3-dioxepane (**7-CA**),[1] 2-methoxyethyl vinyl ether (MOVE),[2] and the HCl adduct of IBVE (**1**)[3] were synthesized according to the literature. Toluene (KANTO, >99.5%; H_2O <10 ppm), *n*-hexane, diethyl ether, and dichloromethane (KANTO, >99.5%; H_2O < 10 ppm) were dried and deoxygenized by passing through columns of a Glass Contour systems before use.

Synthesis of 2-methyl-1,3-oxathiepane (7-CTA)

2-Methyl-1,3-oxathiepane (**7-CTA**) was synthesized by the following procedure. 4-(Tosyloxy)butyl vinyl ether was first synthesized by the reaction between *p*-toluenesulfonyl chloride and 4-hydroxybutyl vinyl ether. A solution of 4-hydroxybutyl vinyl ether (34.0 mL, 275 mmol) in THF (200 mL) was slowly added to a mixture of *p*-toluenesulfonyl chloride (47.7 g, 250 mmol) and triethylamine (77.0 mL, 555 mmol) in a 500 mL flask at 20 °C. After 48 h, the product was extracted with $CHCl_3$ and washed with water. The solvent was removed under reduced pressure to give 4-(tosyloxy)butyl vinyl ether (67.6 g, 250 mmol, yield = 99%).

Then, thioacetylation of 4-(tosyloxy)butyl vinyl ether was conducted by the reaction between 4-(tosyloxy)butyl vinyl ether and potassium thioacetate. A solution of 4-(tosyloxy)butyl vinyl ether (67.6 g, 250 mmol) in $CHCl_3$ (37 mL) was slowly added to another solution of potassium thioacetate (40.7 g, 360 mmol) in DMF (330 mL) in a 500 mL flask at 0 °C. After 2 h, the product was extracted with Et_2O and washed with water. The solvent was removed by evaporation to give 4-(acetylthio)butyl vinyl ether (40.5 g, 232 mmol, yield = 93%).

Finally, **7-CTA** was synthesized by deprotection of the acetyl groups with butylamine, followed by cationic intramolecular cyclization under dilution conditions. The deprotection was conducted by addition of *n*-butylamine (6.0 mL, 60 mmol) to 4-

(acetylthio)butyl vinyl ether (10.4 g, 60.0 mmol) in a 2 L flask at 0 °C. After 14 h, 940 mL of CH_2Cl_2 was added to the reaction solution. Then, cationic intramolecular cyclization was initiated by dropwise addition of a solution of benzenesulfonic acid (240 mL of 50.0 mM CH_2Cl_2 solution, 12.0 mmol) in CH_2Cl_2 (300 mL) to the reaction solution at 0 °C. After 2 h, the reaction was quenched with triethylamine (10 mL). The solvent was removed under vacuum. The residue was extracted with Et_2O and washed with 1 N NaOH aqueous solution and water. Then, the solvent was removed under vacuum to give the crude product. After purification by distillation (3000 Pa, bp = 74 °C), **7-CTA** was obtained as a colorless liquid (3.40 g, 25.7 mmol, yield = 43%). ^{1}H NMR ($CDCl_3$, r.t.): δ 1.51 (d, 3H, CH_3CH, J = 6.4 Hz), 1.70-1.90 (m, 2H, SCH_2CH_2), 1.94 (m, 2H, OCH_2CH_2), 2.69 and 2.80 (dt, 2H, SCH_2CH_2, J_{vic} = 5.2, 6.0, and 8.8 Hz, J_{gem} = –14.0 Hz), 3.75 and 4.02 (ddd, 2H, OCH_2, J_{vic} = 2.4, 6.8, and 8.8 Hz, J_{gem} = –12.2 Hz), 4.95 (q, 1H, CH_3CH, J = 6.4 Hz). ^{13}C NMR ($CDCl_3$, r.t.): δ 23.71 (CH_3), 30.08 (SCH_2CH_2), 30.74 (OCH_2CH_2), 31.15 (SCH_2), 68.17 (OCH_2), 81.48 (CH_3CH).

Synthesis of 2-Methyl-1,3-oxathiolane (5-CTA)

2-Methyl-1,3-oxathiolane (**5-CTA**) was synthesized by a reaction between acetaldehyde dimethyl acetal and 2-mercaptoethanol. Acetaldehyde diethyl acetal (47.5 mL, 450 mmol) was added to a solution containing *p*-toluenesulfonic acid (2.12 g, 11.0 mmol), 2-mercaptoethanol (21.0 mL, 300 mmol), and CH_2Cl_2 (210 mL) in a 500 mL flask at 20 °C. After 24 h, the reaction was quenched with triethylamine (10 mL). The solution was washed with saturated aqueous $NaHCO_3$ solution and H_2O. The solvent was removed by evaporation to give the crude product. After purification by distillation (15400 Pa, bp = 66 °C), **5-CTA** was obtained as a colorless liquid (11.5 g, 110 mmol, yield = 37%). ^{1}H NMR (CD_3OD, r.t.): δ 1.59 (d, 3H, CH_3, J = 6.0 Hz), 3.08 (m, 2H, SCH_2), 3.78 and 4.36 (m, 2H, OCH_2), 5.18 (q, 1H, CH_3CH, J = 6.0 Hz). ^{13}C NMR (CD_3OD, r.t.): δ 22.18 (CH_3), 33.52 (SCH_2), 71.38 (OCH_2), 82.62 (CH_3CH).

Synthesis of 2-Methyl-1,3-oxathiane (6-CTA)

2-Methyl-1,3-oxathiane (**6-CTA**) was synthesized by the reaction between acetaldehyde diethyl acetal and 3-mercapto-1-propanol. Acetaldehyde diethyl acetal (57.0 mL, 400 mmol) was added to a solution containing *p*-toluenesulfonic acid (1.27 g, 6.70 mmol), 3-mercapto-1-propanol (24.0 mL, 268 mmol), and CH_2Cl_2 (590 mL) in a 1 L flask at 20 °C. After 16 h, the reaction was quenched with triethylamine (10 mL). The reaction solution was washed with saturated aqueous $NaHCO_3$ solution and H_2O. The solvent was removed by evaporation to afford the crude product. After purification by

distillation (1400 Pa, bp = 47 °C), **6-CTA** was obtained as a colorless liquid (12.9 g, 109 mmol, yield = 41%). ^{1}H NMR (CD_3OD, r.t.): δ 1.46 (d, 3H, CH_3, J = 6.0 Hz), 1.60 and 1.95 (m, 2H, $CH_2CH_2CH_2$), 2.74 and 3.05 (m, 2H, SCH_2), 3.62 and 4.16 (m, 2H, OCH_2), 4.85 (q, 1H, CH_3CH, J = 6.0 Hz). ^{13}C NMR ($CDCl_3$, r.t.): δ 22.08 (CH_3), 25.51 ($OCH_2CH_2CH_2S$), 27.95 (SCH_2), 69.98 (OCH_2), 78.95 (CH_3CH).

Cationic Copolymerization of EVE and 7-CTA

Cationic copolymerization of EVE and **7-CTA** was carried out by the syringe technique under dry nitrogen in a baked glass tube equipped with a three-way stopcock. A typical example of the polymerization procedure is given below. The cationic copolymerization was initiated by sequential additions of **1** (0.20 mL of 200 mM in toluene solution, 40 μmol) and $ZnCl_2$ (0.20 mL of 40 mM in Et_2O solution, 8.0 μmol) via a dry syringe into a monomer solution including EVE (0.77 mL, 8.04 mmol), **7-CTA** (0.19 mL of 2.16 M CH_2Cl_2 solution, 0.41 mmol), and 1,2-dichlorobenzene (0.18 mL) as an internal standard in CH_2Cl_2 (0.46 mL) at –40 °C. At predetermined intervals, the polymerization was terminated with methanol (2.0 mL) containing a small amount of triethylamine. The monomer conversion was determined from the concentration of residual monomer measured by ^{1}H NMR with 1,2-dichlorobenzene as an internal standard (120 min, 97% for EVE and >99% for **7-CTA**). The quenched reaction mixture was washed with dilute hydrochloric acid and distilled water to remove residual catalyst, evaporated to dryness under reduced pressure, and vacuum-dried to afford the product polymers (M_n = 19500, M_w/M_n = 1.66).

Degradation of Poly(EVE-*co*-7-CTA)

The degradation of poly(EVE-*co*-**7-CTA**) was carried out by the syringe technique under dry nitrogen in a baked glass tube equipped with a three-way stopcock. A typical example of the reaction procedure is given below. The degradation reaction was initiated by the addition of $AgNO_3$ solution (0.30 mL of 1.00 M aqueous solution, 0.30 mmol) via a dry syringe into a polymer solution containing poly(EVE-*co*-**7-CTA**) (40.4 mg, thioacetal: 29.1 μmol, M_n = 22000, M_w/M_n = 1.59) in THF (5.6 mL) at 20 °C. After 3 h, the reaction was quenched by dilution with *n*-hexane. The quenched reaction mixture was washed with dilute hydrochloric acid and distilled water to remove residual catalyst, evaporated to dryness under reduced pressure, and vacuum-dried to afford the product polymers (M_n = 2000, M_w/M_n = 1.31).

Measurement

^{1}H and ^{13}C NMR spectra were recorded on a JEOL ECS-400 spectrometer operated at 400 MHz. The number-average molecular weight (M_n) and the molecular weight distribution (M_w/M_n) of the product polymer were determined by size-exclusion chromatography (SEC) in $CHCl_3$ at 40 °C on two polystyrene gel columns [Shodex K-805 L (pore size: 20–1000 Å; 8.0 mm i.d. × 30 cm) × 2] connected to a JASCO PU-2080 precision pump and JASCO RI-2031 detector. The columns were calibrated against 10 standard polystyrene samples (Agilent Technologies; M_p = 575–2783000, M_w/M_n = 1.02–1.23).

References

1. Maruyama, K.; Kanazawa, A.; Aoshima, S. Controlled Cationic Copolymerization of Vinyl Monomers and Cyclic Acetals via Concurrent Vinyl-Addition and Ring-Opening Mechanisms: The Systematic Study of Structural Effects on The Copolymerization Behavior. *Polym. Chem.,* **2019**, *10*, 5304–5314.
2. Aoshima, S.; Oda, H.; Kobayashi, E. Synthesis of Thermally-Induced Phase Separating Polymer with Well-Defined Polymer Structure by Living Cationic Polymerization. I. Synthesis of Poly(vinyl Ether)s with Oxyethylene Units in the Pendant and Its Phase Separation Behavior in Aqueous Solution. *J. Polym. Sci.: Part A: Polym. Chem.* **1992**, *30*, 2407–2413.
3. Kamigaito, M.; Maeda, Y.; Sawamoto, M.; Higashimura, T. Living Cationic Polymerization of Isobutyl Vinyl Ether by Hydrogen Chloride/Lewis Acid Initiating Systems in the Presence of Salts: In-situ Direct NMR Analysis of the Growing Species. *Macromolecules* **1993**, *26*, 1643–1649.

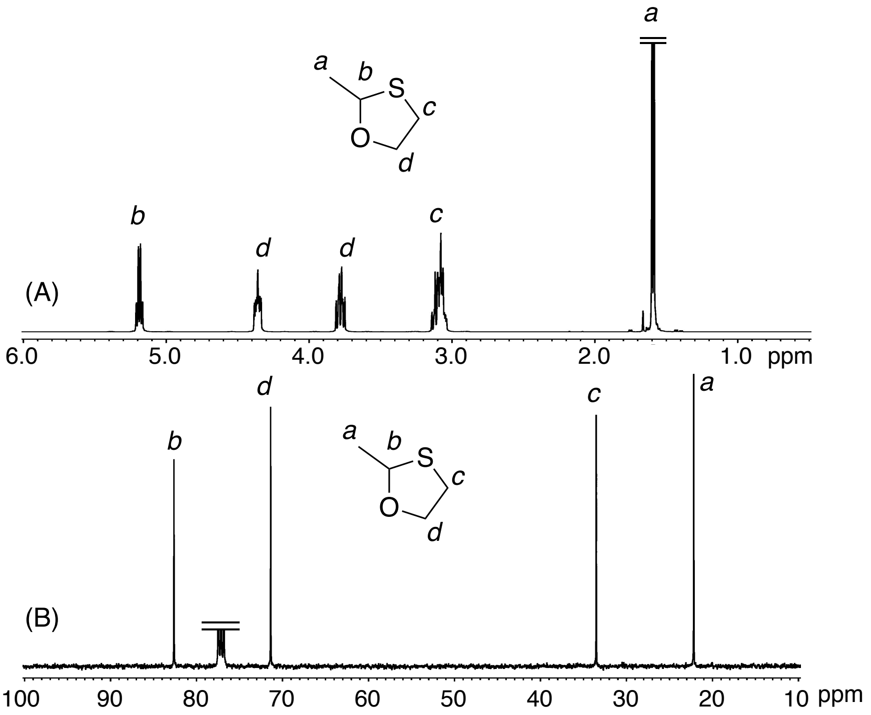

Figure S1. ^{1}H (A) and ^{13}C NMR (B) spectra ($CDCl_3$, r.t.) of **5-CTA**.

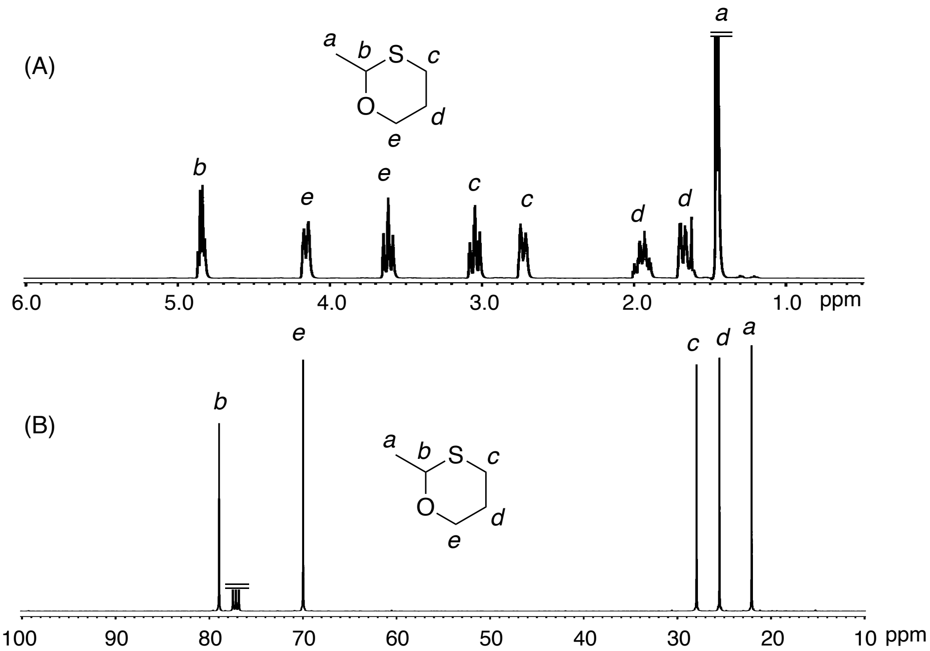

Figure S2. ^{1}H and ^{13}C NMR spectra ($CDCl_3$, r.t.) of **6-CTA**.

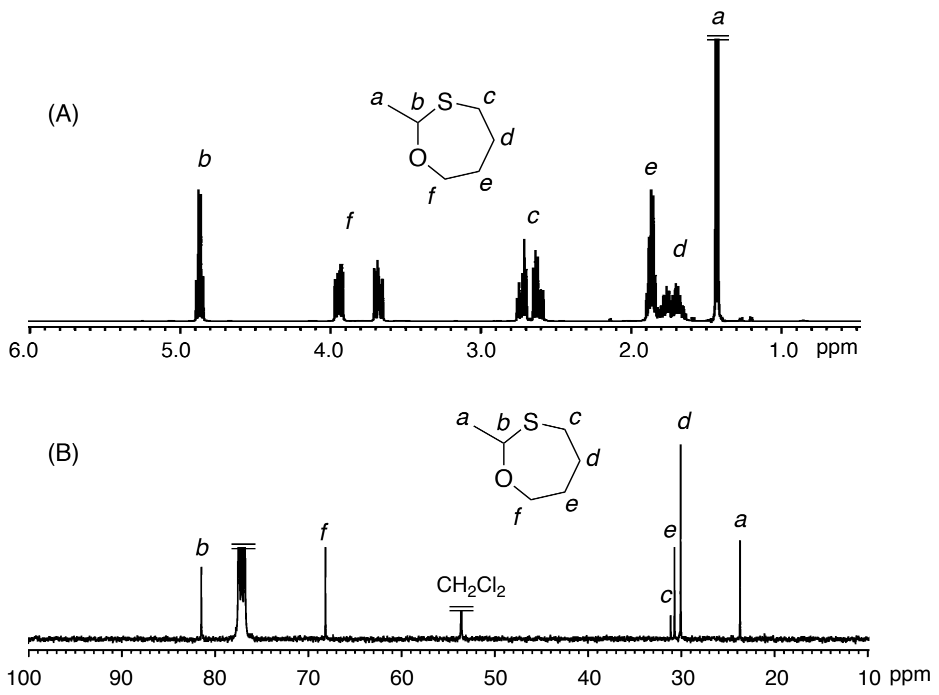

Figure S3. ^{1}H (A) and ^{13}C NMR (B) spectra ($CDCl_3$, r.t.) of **7-CTA**.

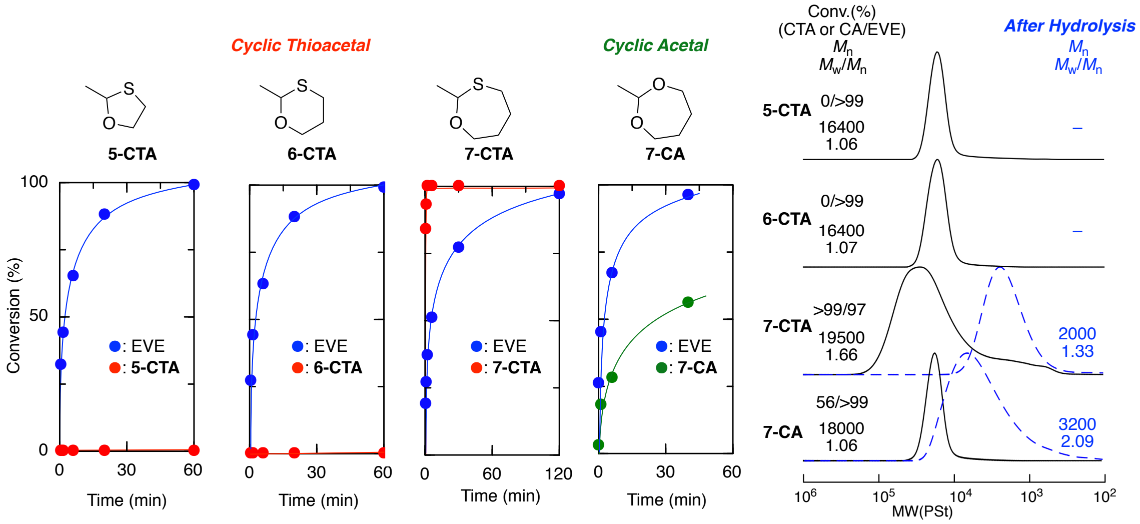

Figure S4. Controlled cationic copolymerization of EVE and various cyclic thioacetals and acetal: $[EVE]_0$/[cyclic thioacetal or cyclic acetal]$_0$/[**1**]$_0$/$[ZnCl_2]_0$ = 4000/200/20/4.0 mM in CH_2Cl_2/*n*-hexane/Et_2O (20/10/10) at –40 °C. Subsequent hydrolysis of the obtained polymers: [thioacetal unit]$_0$/$[AgNO_3]_0$ = 5.0/50 mM in THF/H_2O at 20 °C. [acetal unit]$_0$/[PTSA]$_0$ = 5.0/50 mM in THF/H_2O at 20 °C.

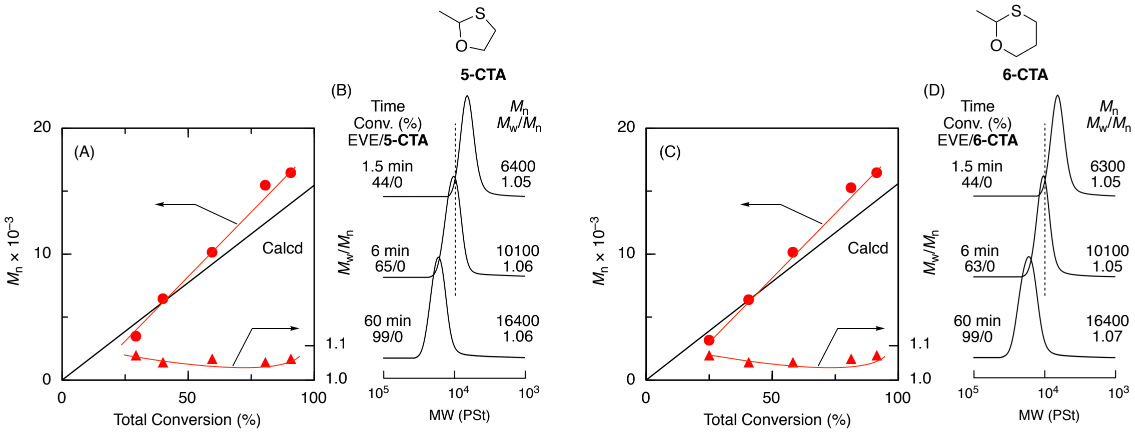

Figure S5. M_n (A, C) and SEC curves (B, D) of the polymers obtained in controlled cationic copolymerization of EVE with **5-CTA** (A, B) or **6-CTA** (C, D): $[EVE]_0$/[**5-CTA** or **6-CTA**]$_0$/[**1**]$_0$/$[ZnCl_2]_0$ = 4000/200/20/4.0 mM in CH_2Cl_2/*n*-hexane/Et_2O (20/10/10) at –40 °C.

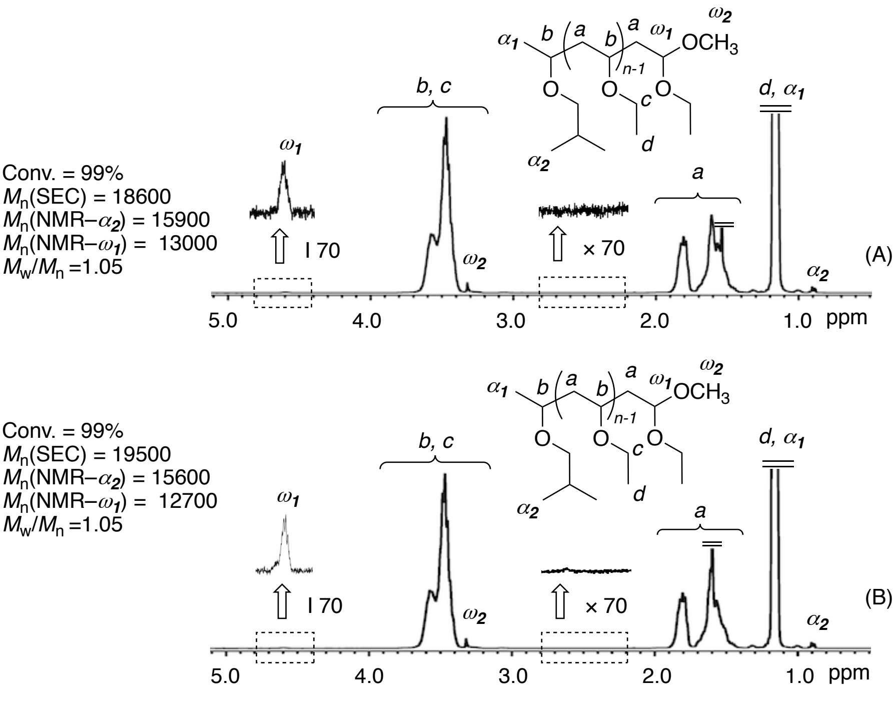

Figure S6. ^{1}H NMR spectra ($CDCl_3$, 55 °C) of the polymers obtained in controlled cationic copolymerization of EVE with **5-CTA** (A) or **6-CTA** (B): $[EVE]_0$/[**5-CTA** or **6-CTA**]$_0$/[**1**]$_0$/$[ZnCl_2]_0$ = 4000/200/20/4.0 mM in CH_2Cl_2/*n*-hexane/Et_2O (20/10/10) at –40 °C.

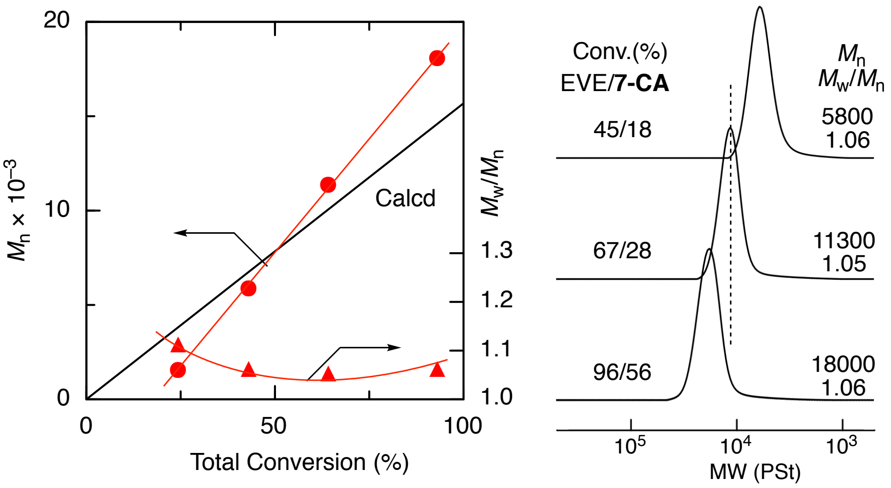

Figure S7. M_n and SEC curves of the polymers obtained in controlled cationic copolymerization of EVE and **7-CA**: [EVE]$_0$/[**7-CA**]$_0$/[**1**]$_0$/[$ZnCl_2$]$_0$ = 4000/200/20/4.0 mM in CH_2Cl_2/*n*-hexane/Et_2O (20/10/10) at –40 °C.

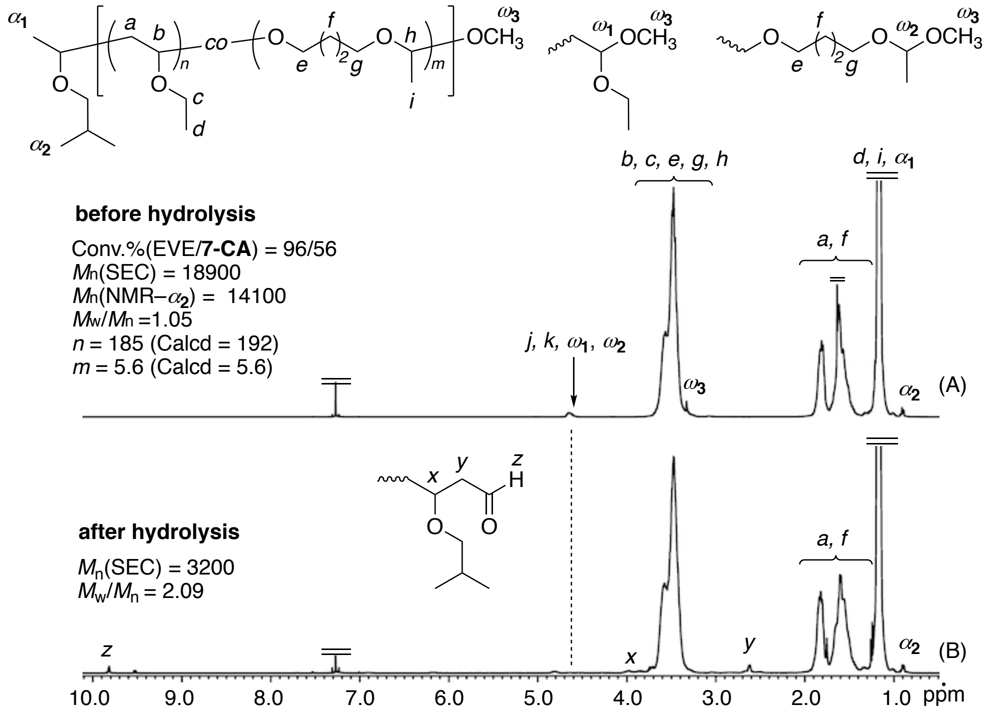

Figure S8. ^{1}H NMR spectra ($CDCl_3$, 55 °C) of the polymers obtained in controlled cationic copolymerization of EVE and **7-CA** before (A) and after (B) hydrolysis: [EVE]$_0$/[**7-CA**]$_0$/[**1**]$_0$/[$ZnCl_2$]$_0$ = 4000/200/20/4.0 mM in CH_2Cl_2/*n*-hexane/Et_2O (20/10/10) at –40 °C. [acetal units]$_0$/[PTSA]$_0$ = 5.0/50 mM in THF/H_2O at 20 °C.

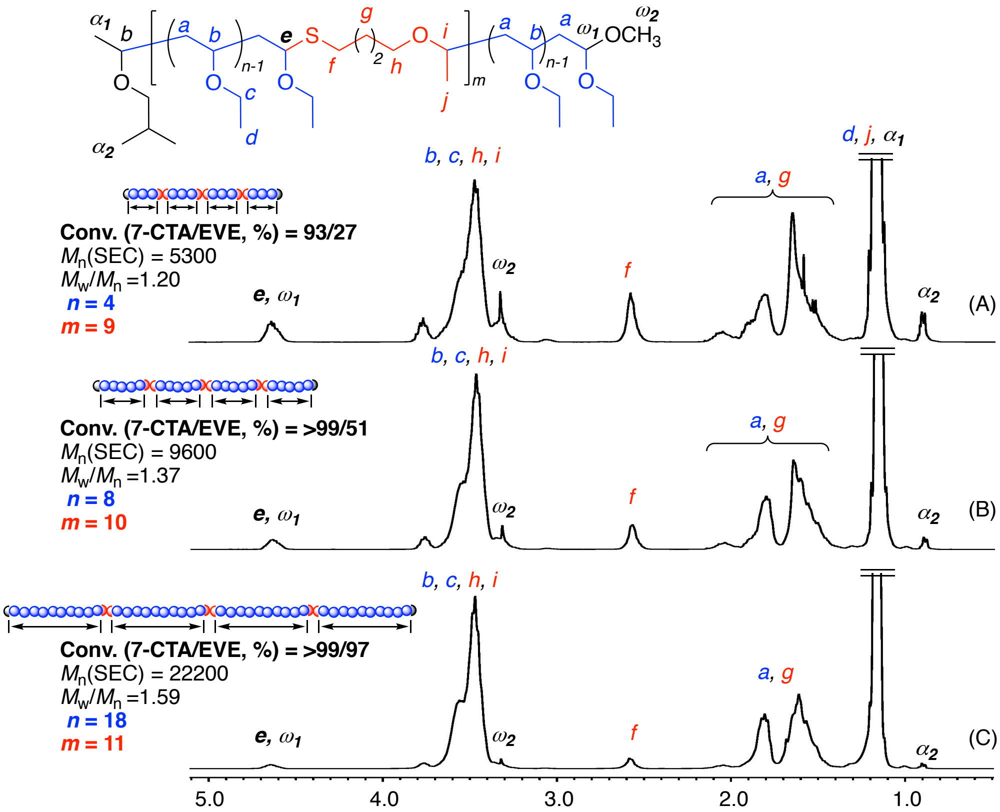

Figure S9. ^{1}H NMR spectra ($CDCl_3$, 55 °C) of the polymers obtained at different **7-CTA**/EVE conversions (A for 93%/27%, B for >99%/51%, C for >99%/97%) in controlled cationic copolymerization of EVE and **7-CTA**.

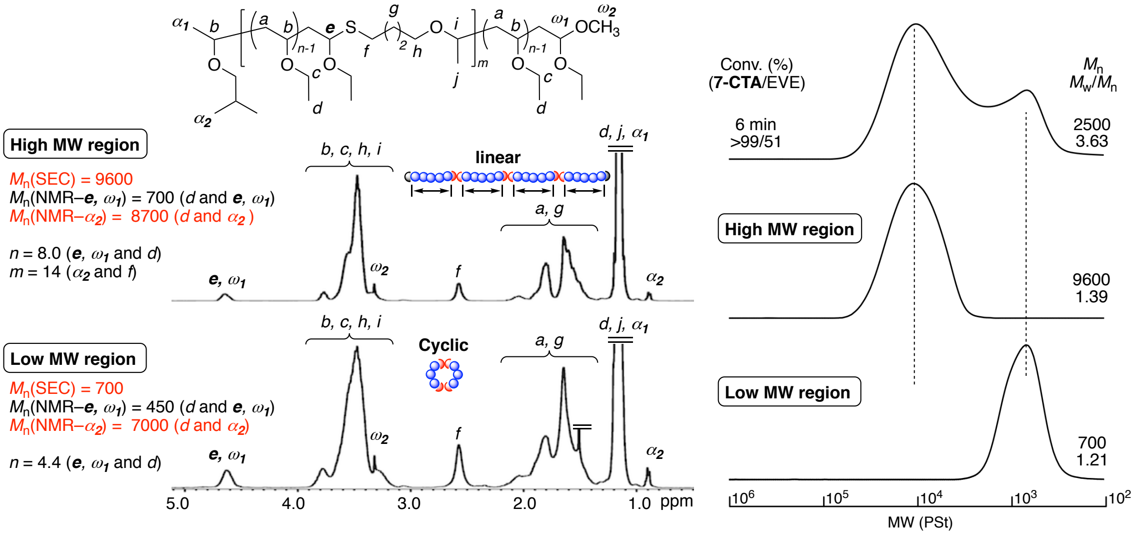

Figure S10. ^{1}H NMR spectra ($CDCl_3$, 55 °C) and SEC curves of the polymers with high and low-molecular-weight regions after separation by preparative SEC.

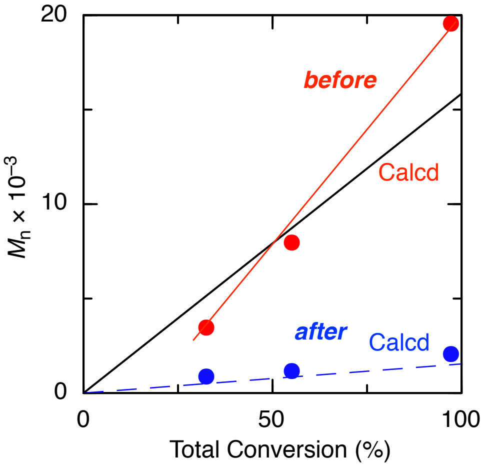

Figure S11. M_n curves of the polymers before and after hydrolysis using an $AgNO_3$ solution: [thioacetal unit]$_0$/[$AgNO_3$]$_0$ = 5.0/50 mM in THF/H_2O at 20 °C.

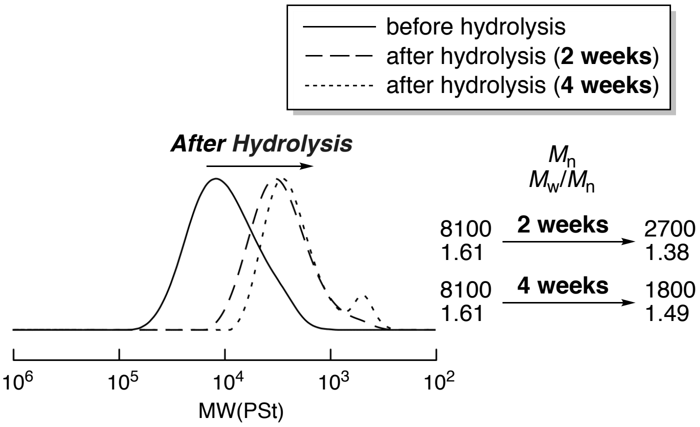

Figure S12. SEC curves of the polymers before and after hydrolysis using a PTSA solution: [thioacetal unit]$_0$/[PTSA]$_0$ = 5.0/50 mM in THF/H_2O at 20 °C.

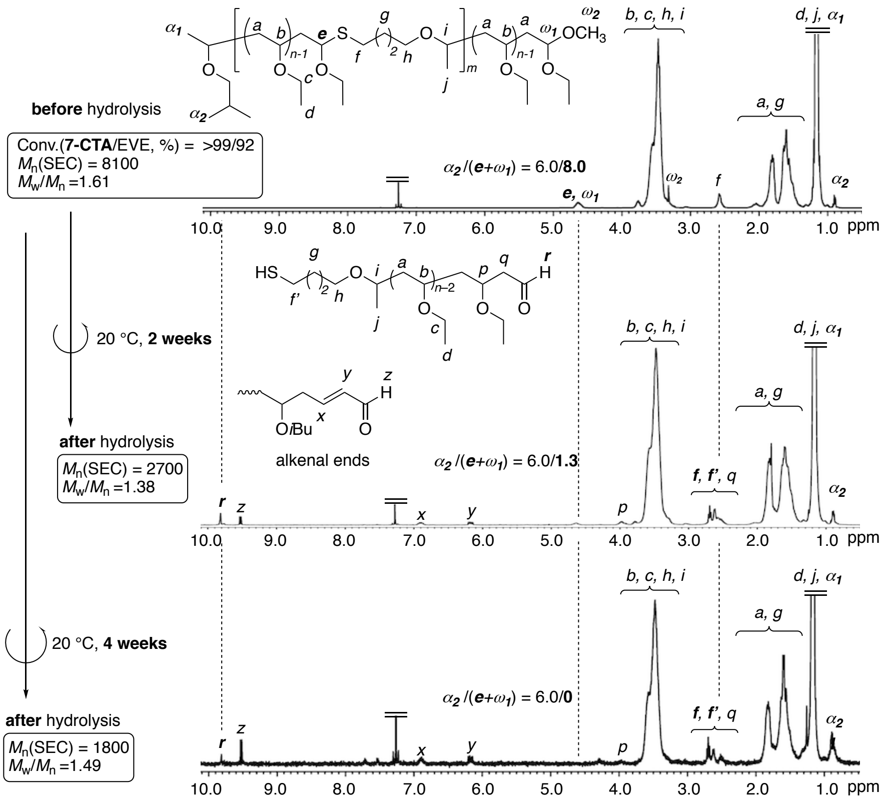

Figure S13. ^{1}H NMR spectra ($CDCl_3$, 55 °C) of the polymers before and after hydrolysis using a PTSA solution: [thioacetal unit]$_0$/[PTSA]$_0$ = 5.0/50 mM in THF/H_2O at 20 °C.

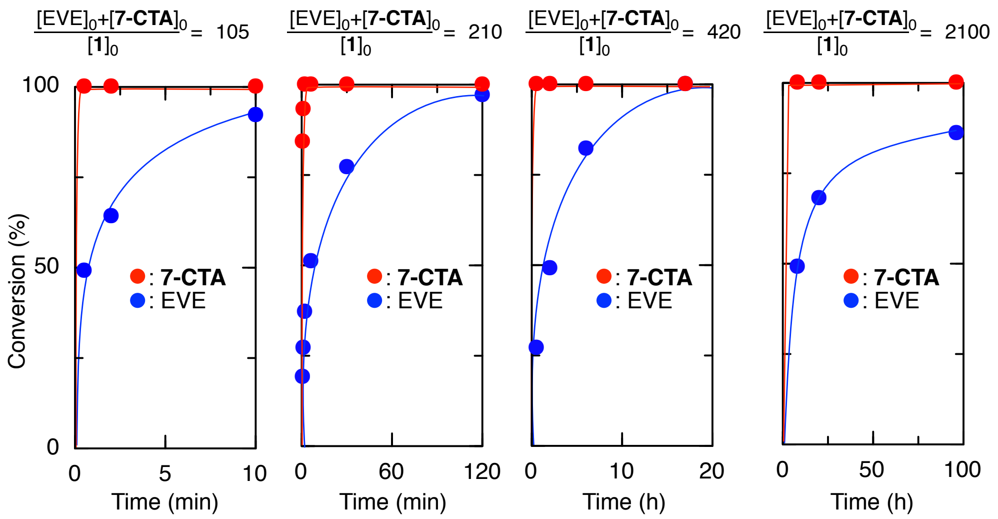

Figure S14. Time-conversion curves in controlled cationic copolymerization of EVE and **7-CTA** at varying the feed ratio of total monomer to **1**: [EVE]$_0$/[**7-CTA**]$_0$/[**1**]$_0$/[$ZnCl_2$]$_0$ = 4000/200/40, 20, 10/4.0 or 6000/300/3.0/6.0 mM in CH_2Cl_2/*n*-hexane/Et_2O (20/10/10) at –40 °C.

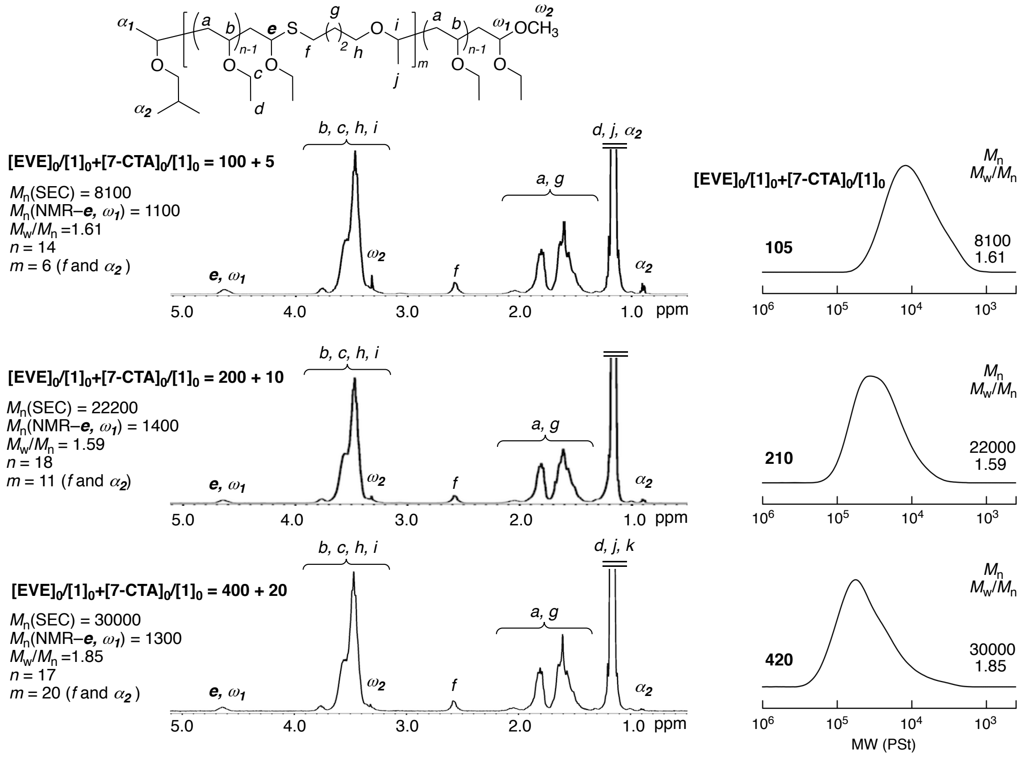

Figure S15. ^{1}H NMR spectra and SEC curves of the polymers obtained in controlled cationic copolymerization of EVE and **7-CTA** for Figure S14 after purification by preparative SEC.

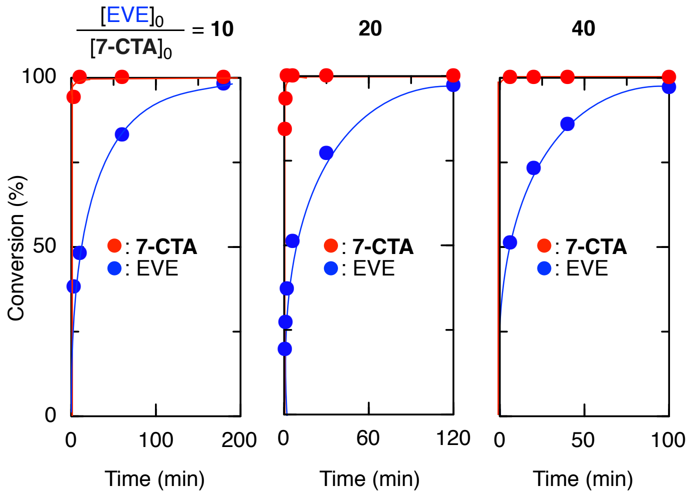

Figure S16. Time-conversion curves in controlled cationic copolymerization of EVE and **7-CTA** at varying the feed ratio of EVE to **7-CTA**: $[EVE]_0/[\mathbf{7\text{-}CTA}]_0/[\mathbf{1}]_0/[ZnCl_2]_0$ = 4000/400, 200, 100/20/4.0 mM in CH_2Cl_2/*n*-hexane/Et_2O (20/10/10) at –40 °C.

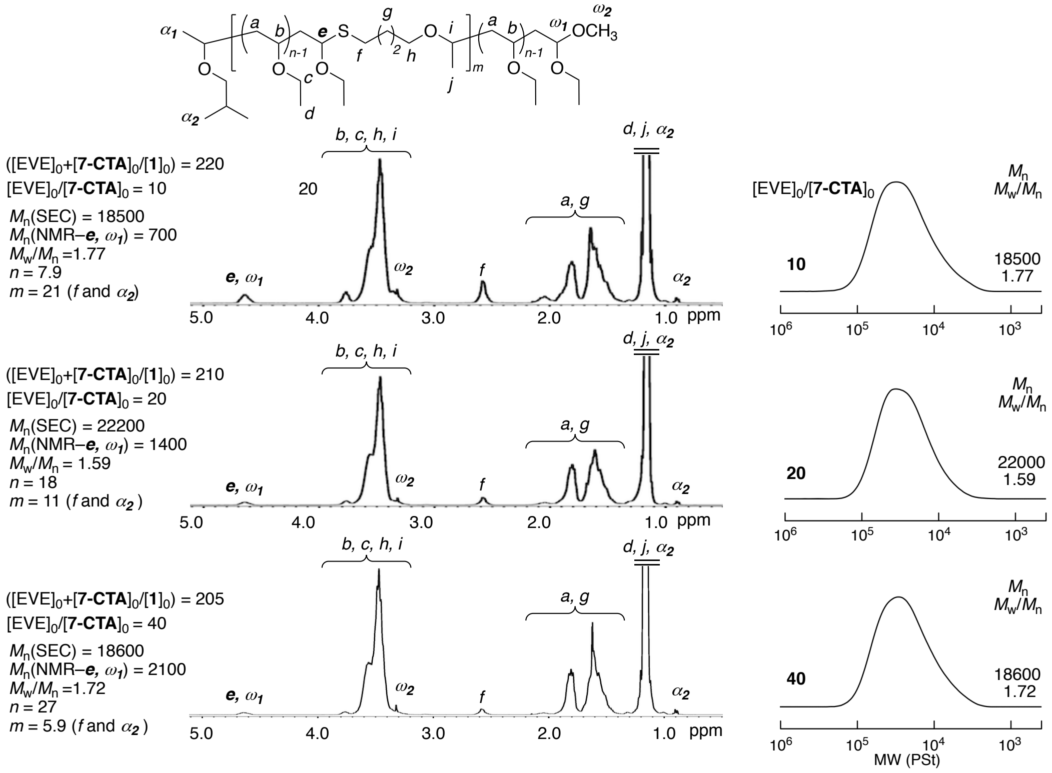

Figure S17. ^{1}H NMR spectra and SEC curves obtained in controlled cationic copolymerization of EVE and **7-CTA** for Figure S16 after purification by preparative SEC.

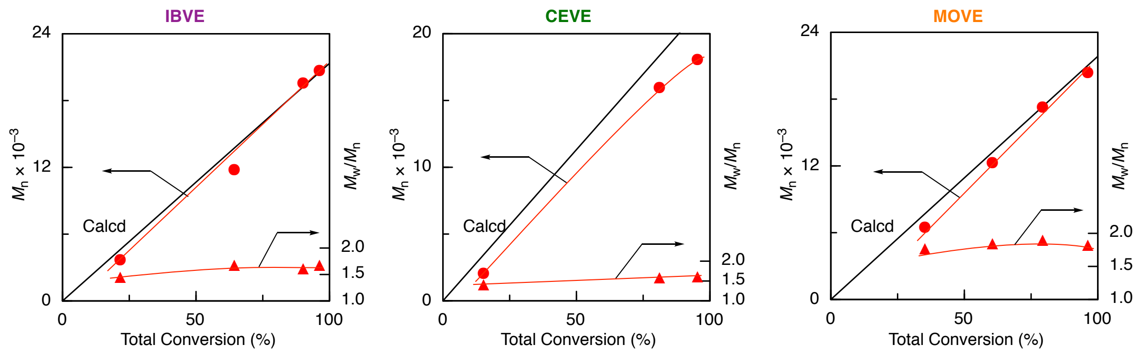

Figure S18. M_n and M_w/M_n values of the polymers obtained in controlled cationic copolymerization of various vinyl ethers with **7-CTA**: $[VE]_0/[\textbf{7-CTA}]_0/[\textbf{1}]_0/[ZnCl_2]_0$ = 4000/200/20/4.0 mM in CH_2Cl_2/*n*-hexane/Et_2O (20/10/10) at –40 °C.

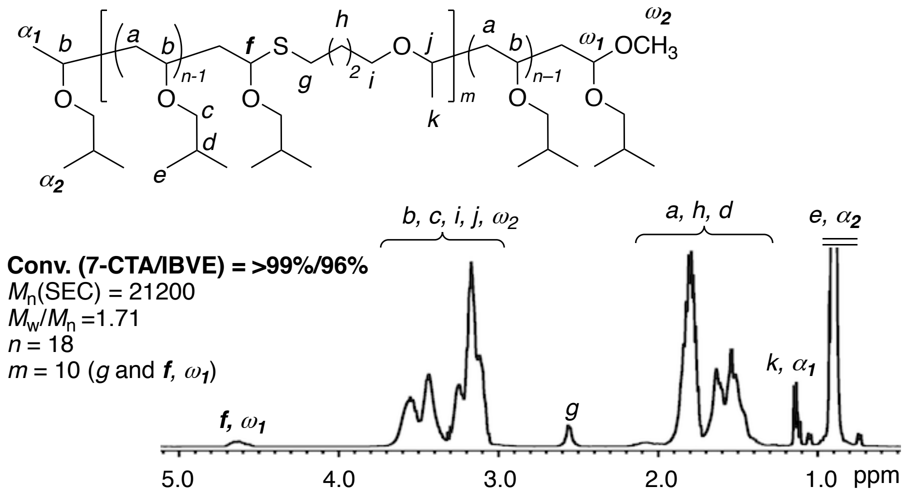

Figure S19. ^{1}H spectrum ($CDCl_3$, 55 °C) of degradable poly(IBVE) obtained in controlled cationic copolymerization of IBVE with **7-CTA**: $[IBVE]_0$/[**7-CTA**]$_0$/[**1**]$_0$/$[ZnCl_2]_0$ = 4000/200/20/4.0 mM in CH_2Cl_2/*n*-hexane/Et_2O (20/10/10) at –40 °C.

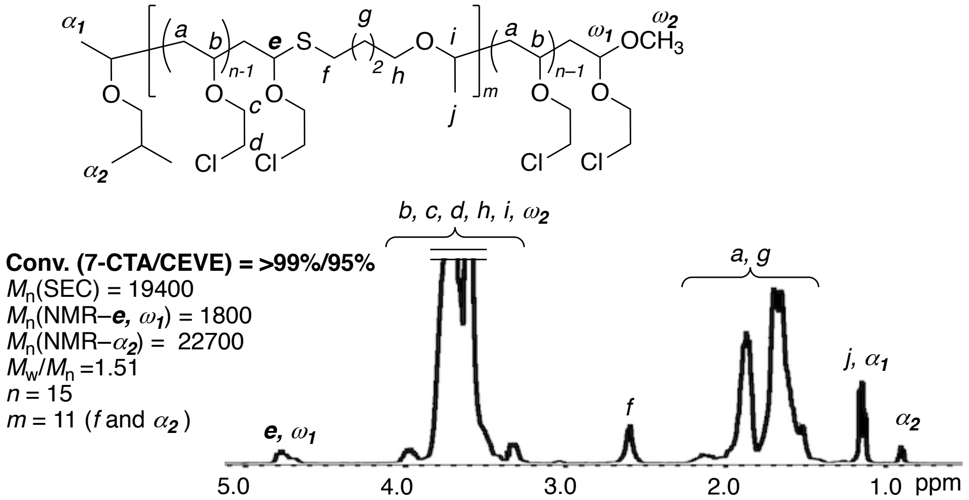

Figure S20. ^{1}H spectrum ($CDCl_3$, 55 °C) of degradable poly(CEVE) obtained in controlled cationic copolymerization of CEVE with **7-CTA**: $[CEVE]_0$/[**7-CTA**]$_0$/[**1**]$_0$/$[ZnCl_2]_0$ = 4000/200/20/4.0 mM in CH_2Cl_2/*n*-hexane/Et_2O (20/10/10) at –40 °C.

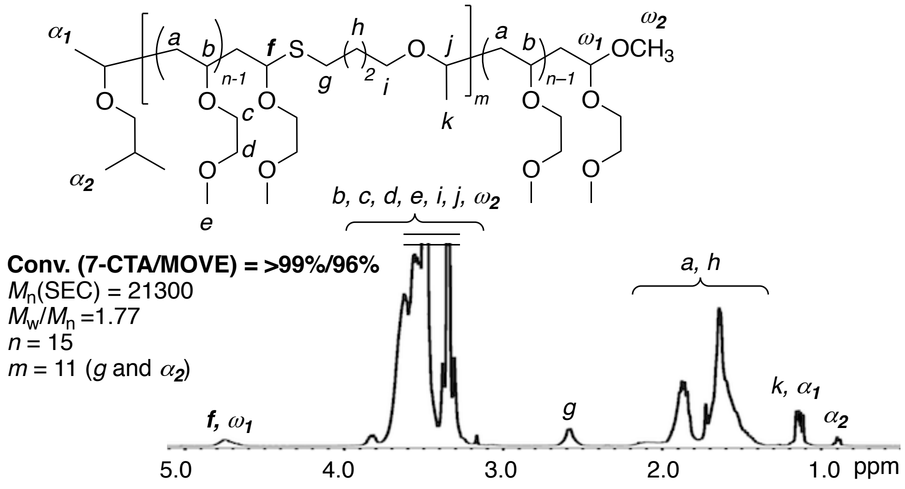

Figure S21. ^{1}H spectrum ($CDCl_3$, 55 °C) of degradable poly(MOVE) obtained in controlled cationic copolymerization of MOVE with **7-CTA**: $[MOVE]_0/[\mathbf{7\text{-}CTA}]_0/[\mathbf{1}]_0/[ZnCl_2]_0$ = 4000/200/20/4.0 mM in CH_2Cl_2/*n*-hexane/Et_2O (20/10/10) at –40 °C.

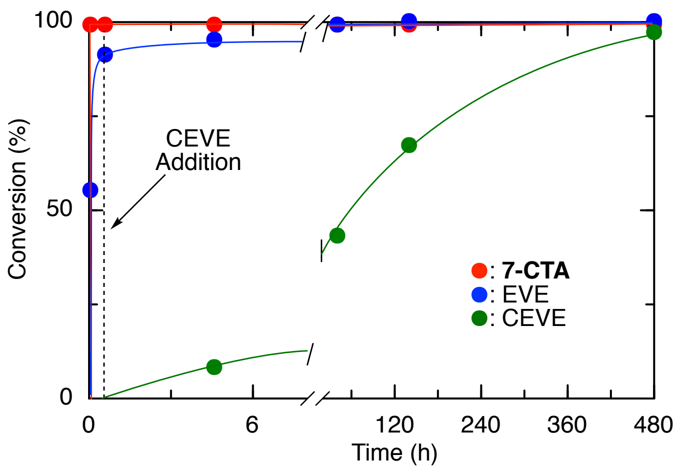

Figure S22. Time-conversion curves for one-pot synthesis of degradable multiblock copolymers of EVE and CEVE by controlled cationic copolymerization with **7-CTA**: $[EVE]_0/[CEVE]_{add}/[\mathbf{7\text{-}CTA}]_0/[\mathbf{1}]_0/[ZnCl_2]_0$ = 2000/2000/20/4.0 mM in CH_2Cl_2/*n*-hexane/Et_2O (20/10/10) at –40 °C.

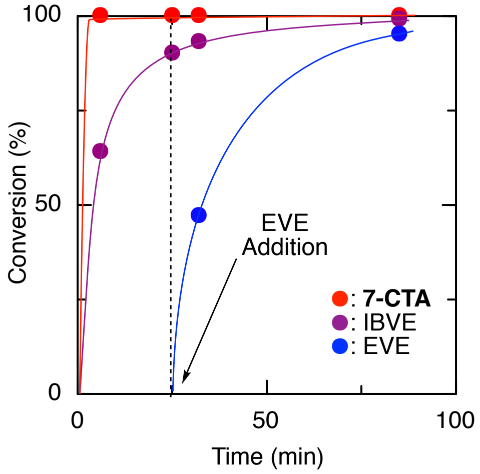

Figure S23. Time-conversion curves for one-pot synthesis of degradable multiblock copolymers of IBVE and EVE by controlled cationic copolymerization with **7-CTA**: $[IBVE]_0/[EVE]_{add}/[$**7-CTA**$]_0/[$**1**$]_0/[ZnCl_2]_0$ = 2000/2000/200/20/4.0 mM in CH_2Cl_2/*n*-hexane/Et_2O (20/10/10) at –40 °C.

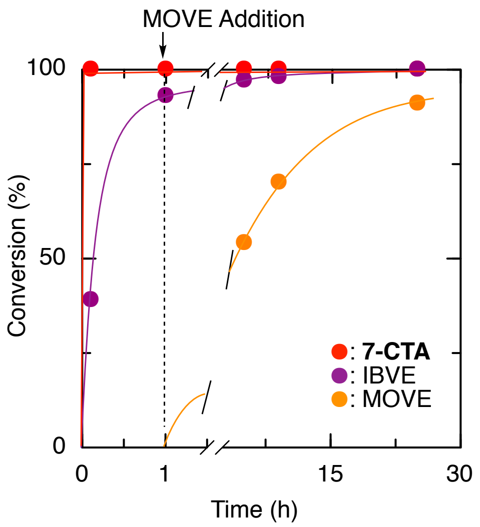

Figure S24. Time-conversion curves for one-pot synthesis of degradable multiblock copolymers of IBVE and MOVE by controlled cationic copolymerization with **7-CTA**: $[IBVE]_0/[MOVE]_{add}/[$**7-CTA**$]_0/[$**1**$]_0/[ZnCl_2]_0$ = 2000/2000/200/20/4.0 mM in CH_2Cl_2/*n*-hexane/Et_2O (20/10/10) at –40 °C.

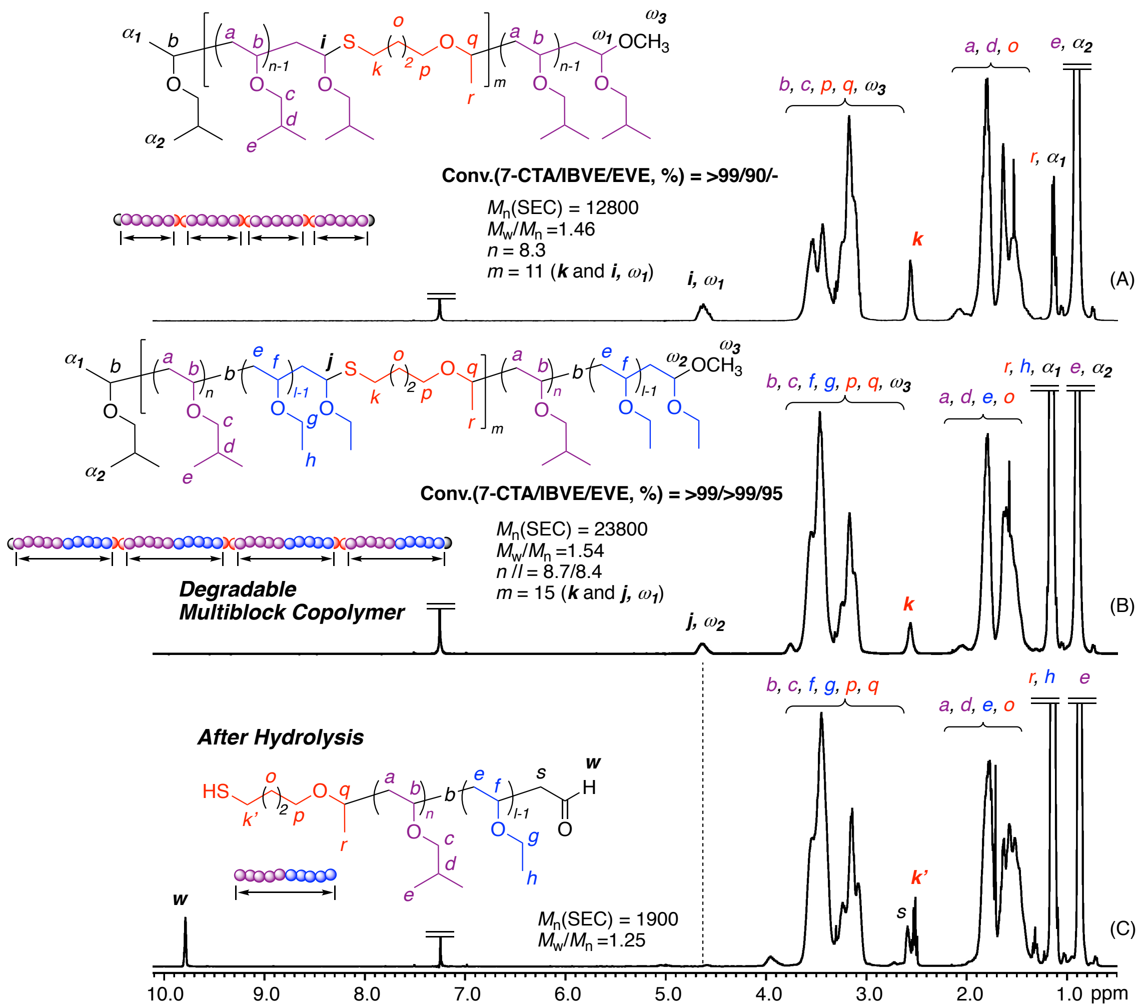

Figure S25. ^{1}H NMR spectra ($CDCl_3$, 55 °C) of degradable poly(EVE) (A), multiblock poly(IBVE-EVE) (B), and the polymers obtained after hydrolysis of multiblock poly(IBVE-EVE) (C).

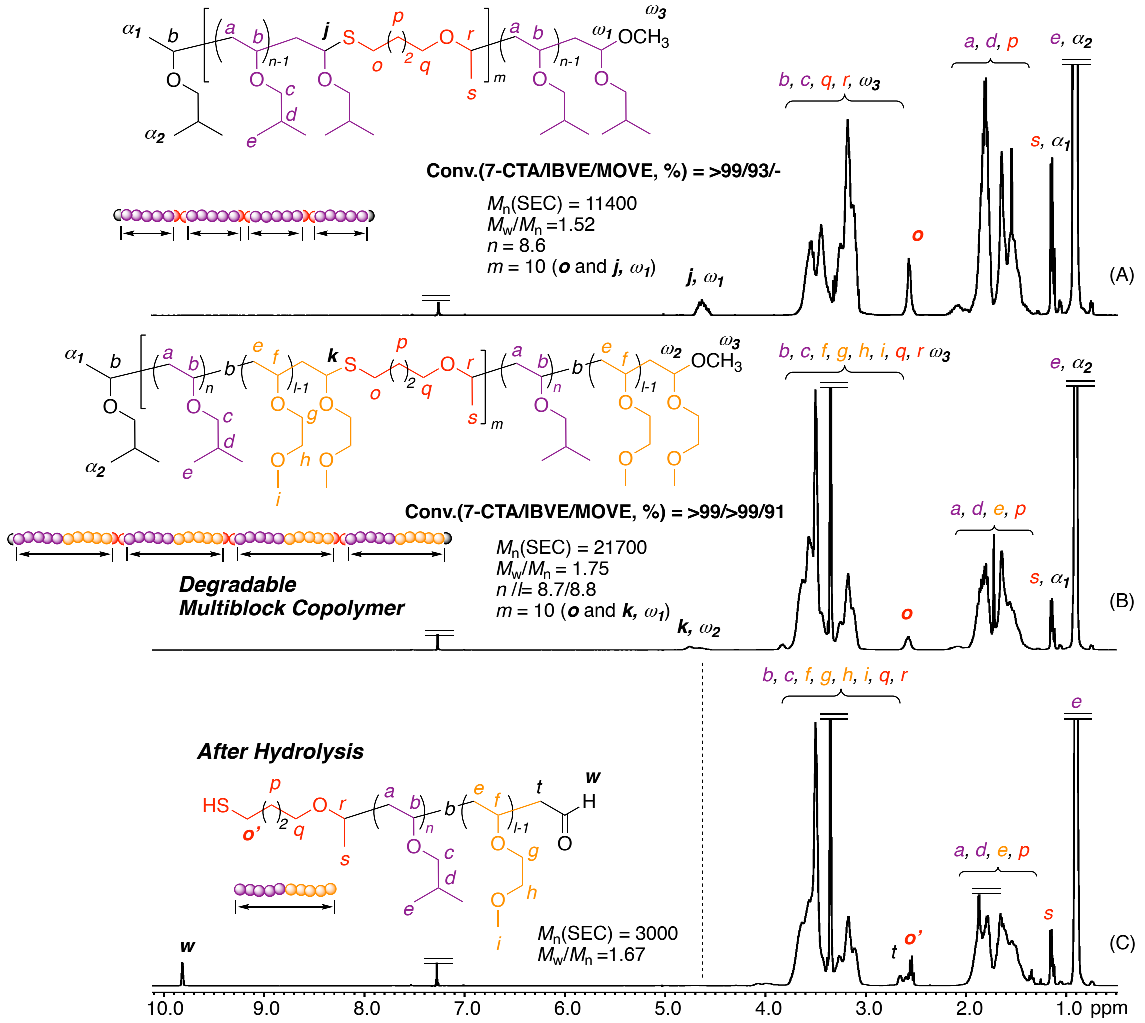

Figure S26. ^{1}H NMR spectra ($CDCl_3$, 55 °C) of degradable poly(IBVE) (A), multiblock poly(IBVE-MOVE) (B), and the polymers obtained after hydrolysis of multiblock poly(IBVE-MOVE) (C).

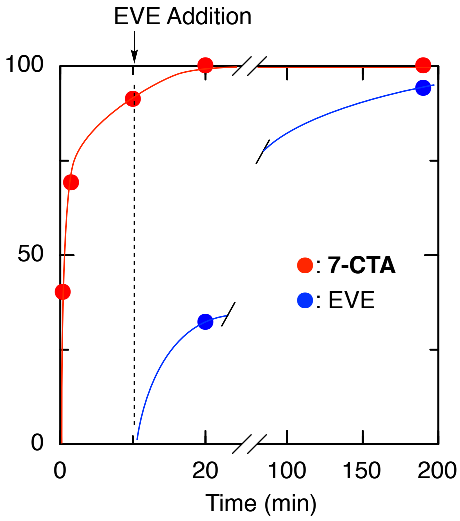

Figure S27. Time-conversion curves for sequential cationic polymerization of **7-CTA** followed by cationic polymerization of EVE: [**7-CTA**]$_0$/[**1**]$_0$/[$ZnCl_2$]$_0$ = 320/32/6.4 mM (before EVE addition), [EVE]$_{add}$/[**7-CTA**]$_0$/[**1**]$_0$/[$ZnCl_2$]$_0$ = 4000/200/20/4.0 mM (after EVE addition), in CH_2Cl_2/*n*-hexane/Et_2O (20/10/10) at –40 °C.

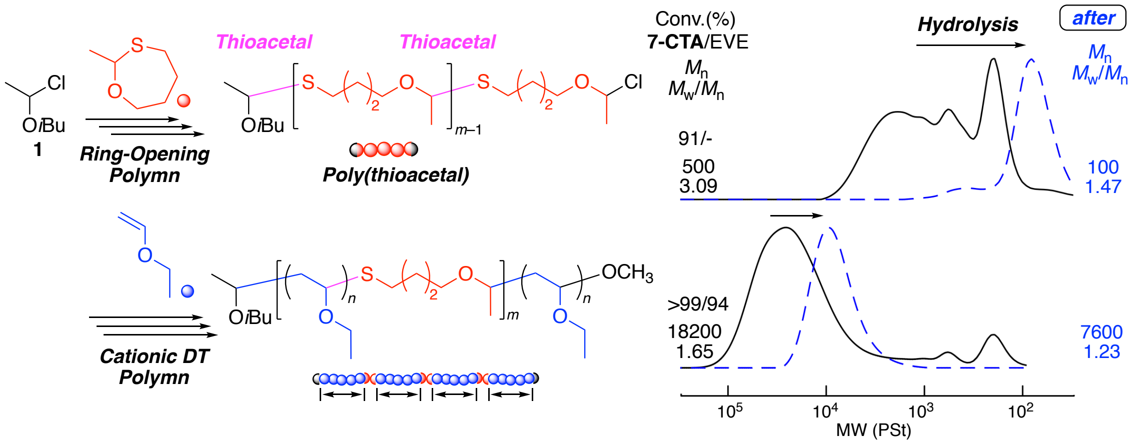

Figure S28. Cationic ring-opening homopolymerization of **7-CTA** and subsequent cationic polymerization of EVE: [**7-CTA**]$_0$/[**1**]$_0$/[$ZnCl_2$]$_0$ = 320/32/6.4 mM (before EVE addition), [EVE]$_{add}$/[**7-CTA**]$_0$/[**1**]$_0$/[$ZnCl_2$]$_0$ = 4000/200/20/4.0 mM (after EVE addition), in CH_2Cl_2/*n*-hexane/Et_2O (20/10/10) at –40 °C.

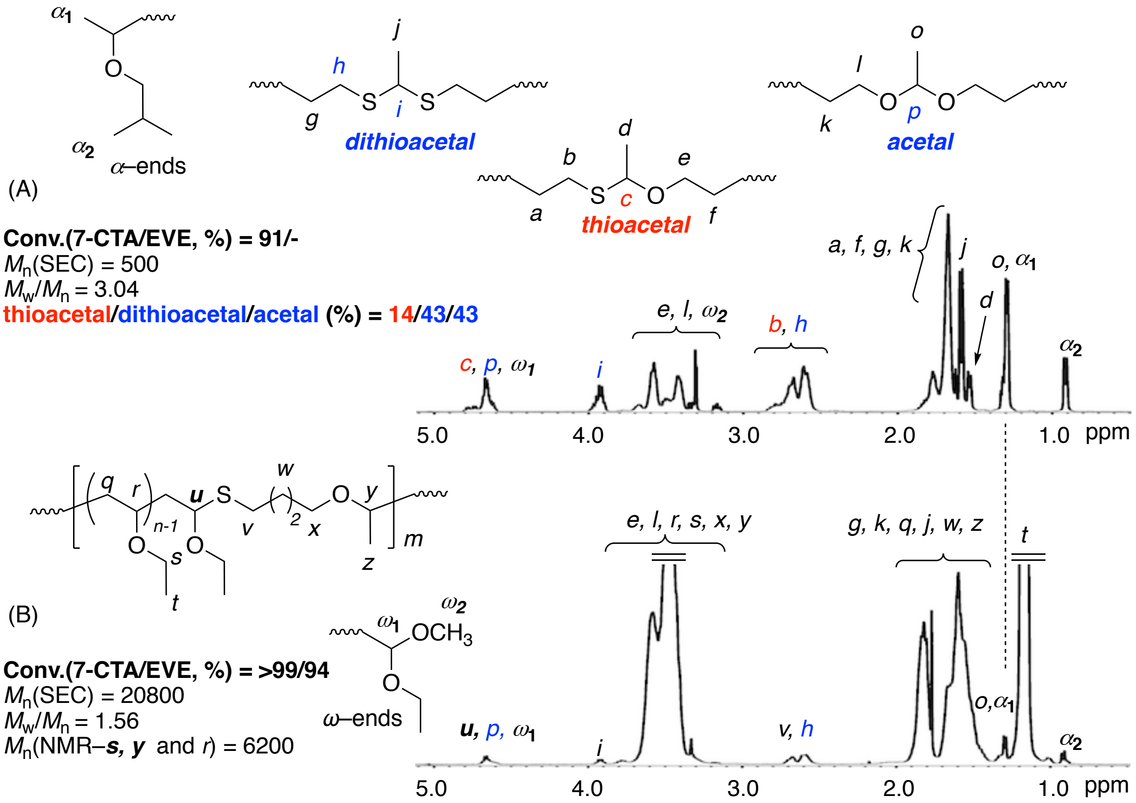

Figure S29. ^{1}H NMR spectra ($CDCl_3$, 55 °C) of poly(**7-CTA**) (A) and the polymers obtained after EVE addition (B).

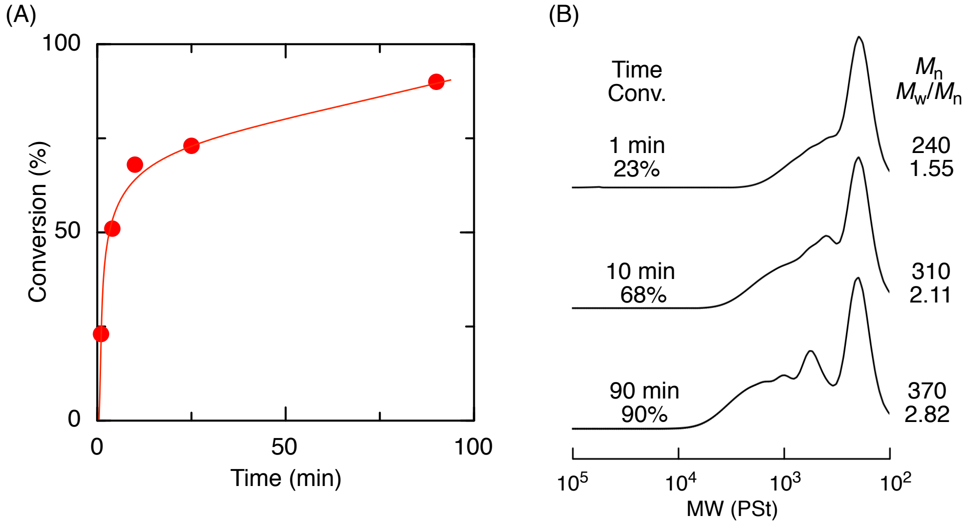

Figure S30. Time-conversion (A) and SEC (B) curves for cationic ring-opening homopolymerization of **7-CTA**: [**7-CTA**]$_0$/[**1**]$_0$/[$ZnCl_2$]$_0$ = 200/20/4.0 mM in CH_2Cl_2 at –40 °C.

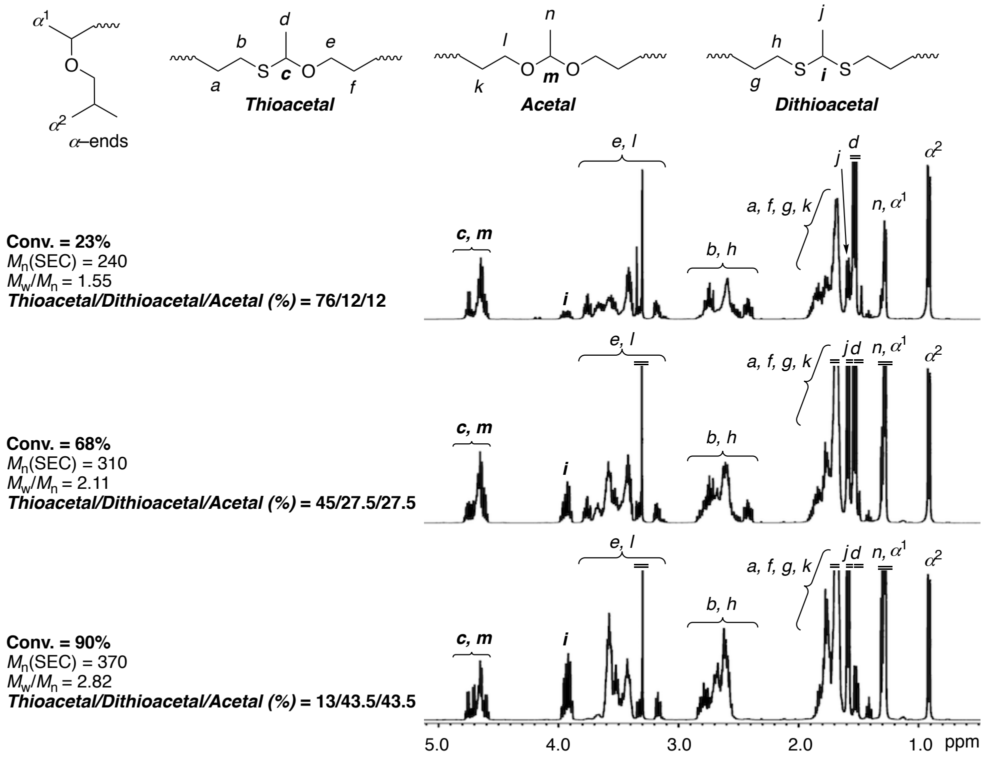

Figure S31. ^{1}H NMR spectra ($CDCl_3$, 55 °C) of the polymers obtained at different **7-CTA** conversions in cationic ring-opening homopolymerization of **7-CTA**.

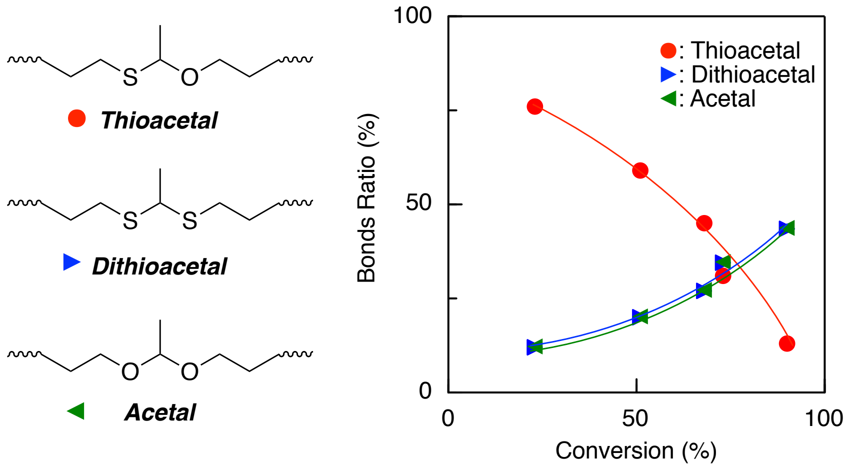

Figure S32. Bond ratios of thioacetal, dithioacetal, and acetal in cationic ring-opening homopolymerization of **7-CTA** at varying the feed ratio of EVE to **7-CTA**: [**7-CTA**]$_0$/[**1**]$_0$/[$ZnCl_2$]$_0$ = 200/20/4.0 mM in CH_2Cl_2 at –40 °C.

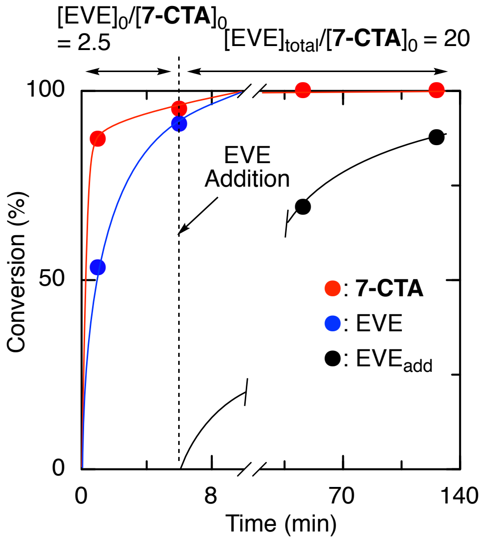

Figure S33. Time-conversion curves for sequential cationic copolymerization of **7-CTA** and a small amount of EVE followed by cationic polymerization of EVE: $[EVE]_0/[\mathbf{7\text{-}CTA}]_0/[\mathbf{1}]_0/[ZnCl_2]_0$ = 750/300/30/6.0 mM (before EVE addition), $[EVE]_{total}/[\mathbf{7\text{-}CTA}]_0/[\mathbf{1}]_0/[ZnCl_2]_0$ = 4000/200/20/4.0 mM (after EVE addition), in CH_2Cl_2/*n*-hexane/Et_2O (20/10/10) at –40 °C.

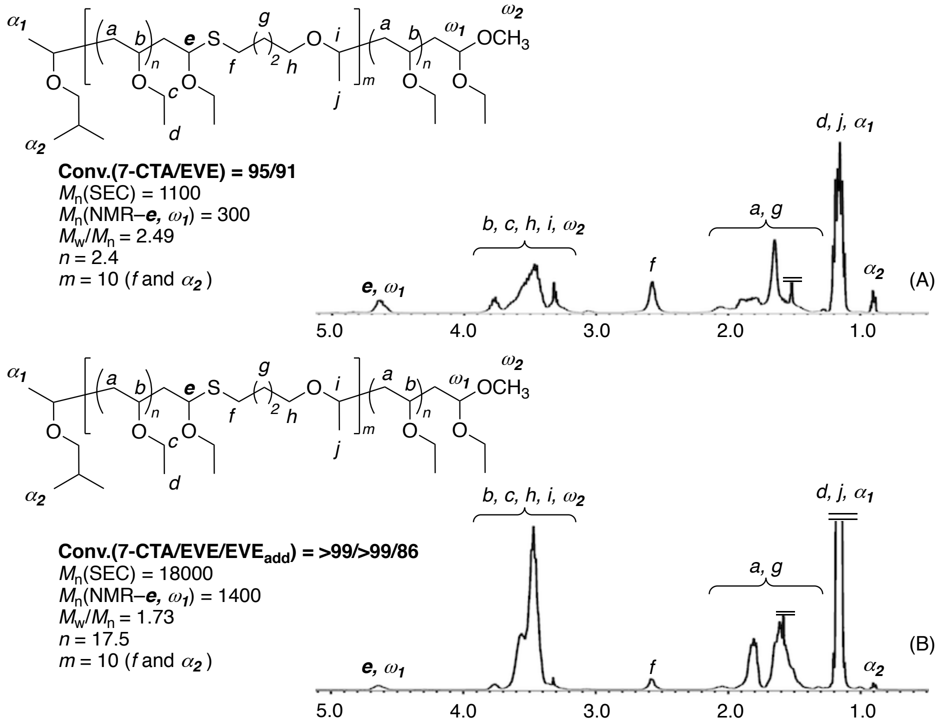

Figure S34. ^{1}H NMR spectra ($CDCl_3$, 55 °C) of the polymers of **7-CTA** with a small amount of EVE (A) and the polymers obtained after EVE addition (B).

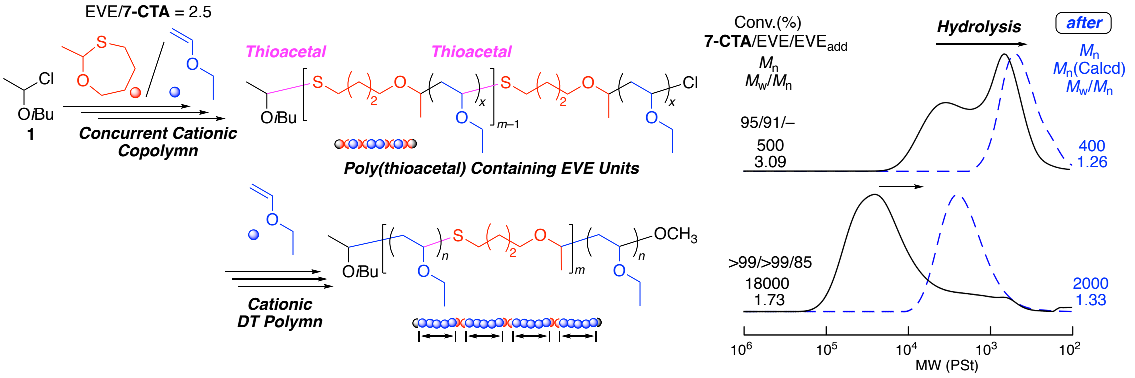

Figure S35. Sequential cationic copolymerization of **7-CTA** and a small amount of EVE and subsequent cationic polymerization of additional EVE: $[EVE]_0$/[**7-CTA**]$_0$/[**1**]$_0$/$[ZnCl_2]_0$ = 750/300/30/6.0 mM (before EVE addition), $[EVE]_{total}$/[**7-CTA**]$_0$/[**1**]$_0$/$[ZnCl_2]_0$ = 4000/200/20/4.0 mM (after EVE addition), in CH_2Cl_2/*n*-hexane/Et_2O (20/10/10) at –40 °C.
